# Grape seed meal by-product is able to counteract oxidative stress induced by lipopolysaccharide and dextran sulphate in IPEC cells and piglets after weaning

**DOI:** 10.1371/journal.pone.0283607

**Published:** 2023-04-13

**Authors:** Gina Cecilia Pistol, Daniela Eliza Marin, Valeria Cristina Bulgaru, Andrei Cristian Anghel, Mihaela Sărăcilă, Mihaela Vlassa, Miuta Filip, Ionelia Taranu

**Affiliations:** 1 Laboratory of Animal Biology, INCDBNA-IBNA, National Research—Development Institute for Animal Biology and Nutrition, Balotesti, Romania; 2 Laboratory of Feed and Food Quality, INCDBNA-IBNA, National Research—Development Institute for Animal Biology and Nutrition, Balotesti, Romania; 3 Raluca Ripan Institute for Research in Chemistry, Babeș-Bolyai University, Cluj-Napoca, Romania; Institute of medical research and medicinal plant studies, CAMEROON

## Abstract

Oxidative stress is a pivotal factor in the pathogenesis of intestinal inflammation, leading to cellular damage and tissue injury. Natural antioxidants compounds found in agro-industrial by-products have proven their effectiveness in treatment of intestinal inflammation and oxidative stress, exhibiting many favourable effects. The aim of this study was to evaluate the capacity of a grape seed meal byproduct (GSM) to counteract the effects induced by *E*. *coli* lipopolysaccharide (LPS, 5μg/ml) *in vitro* on IPEC-1 cells and by dextran sulphate sodium (DSS, 1g/b.w./day) *in vivo* on piglets after weaning. Reactive oxygen species (ROS), pro-oxidant markers (malondialdehyde MDA, thiobarbituric acid reactive substances TBARS, protein carbonyl, DNA oxidative damage) antioxidant enzymes (catalase -CAT, superoxide dismutase -SOD, glutathione peroxidase -GPx, endothelial and inducible nitric oxide synthases -eNOS and iNOS) and several important components of Keap1/Nrf2 signalling pathway were analysed in IPEC-1 cells as well as in piglet’s colon and lymph nodes. Our results demonstrated that GSM extract or 8% dietary GSM showed anti-oxidant properties counteracting the pro-oxidant response (ROS, MDA-TBARS, protein carbonyl, DNA/RNA damage) induced by LPS or DSS and restoring the levels of endogenous antioxidant enzymes, including CAT, SOD, GPx, eNOS and iNOS in colon and mesenteric lymph nodes. These beneficial effects were modulated via Nrf2 signalling pathway in both *in vitro* and *in vivo* studies.

## 1. Introduction

Intestinal inflammatory diseases are chronic, progressive disorders of the gastrointestinal (GI) tract [1]. The pathogenesis of intestinal inflammatory diseases results from a complex interplay between an unbalanced immune system, environmental factors (diet, radiation, heavy metals, pesticides, xenobiotics and antibiotics), intestinal microbiota and genetic variation [2]. Oxidative stress is a pivotal factor in the pathogenesis of intestinal inflammation, leading to cellular damage and tissue injury [1]. It is tightly associated with chronic inflammation and disruption of redox signalling reflecting a disequilibrium between systemic occurrence of ROS and the capacity of biological systems to restore tissue injuries and to detoxify its intermediates [3].

In mucosal tissue, the generation of reactive oxygen species disturbs cellular homeostasis, contributing to cell injury and increasing the permeability of mucosal barrier, accelerating and perpetuating the inflammatory processes. Furthermore, antioxidant enzymes, such as CAT, SOD, and GPx which counteract ROS are deficient in intestinal inflammation [4].

In human, there are limitations in the use of anti-inflammatory and anti-oxidative drugs such as immunosuppressants and corticosteroids because of the adverse effects over prolonged treatment periods [4–6]. In farm animals, inflammation and oxidative stress are a frequent and severe problems especially after the ban of in feed antibiotics utilization. Studies are now focusing on various substances which have both antioxidant and anti-inflammatory properties and which could reduce or totally replace essential drugs. Of these, the natural antioxidants such as polyphenols (resveratrol, curcumin etc.), polyunsaturated fatty acids (omega -3/-6/-9), fibre, etc might be beneficial in the treatment of intestinal inflammation and oxidation, exhibiting many positive effects (immunomodulatory action, scavenging of free radicals, increasing antioxidant defence response) [6–8]. The role of such phytochemicals was also demonstrated in regulating the maintenance of gut homeostasis, mucus function and the impact on gut microbiota in intestinal inflammation [2, 9].

Grape wastes (grape pomace-GP and grape seed meal-GSM) resulted from winery and oil industries are rich in bioactive compounds, containing great amounts of polyphenols from the classes of flavonoids such as anthocyanins, catechins etc, with many recognized health benefits, due to their antioxidant, immunomodulatory and antibacterial properties [10]. Many *in vitro* and *in vivo* studies demonstrated the benefits of grape waste used either individual compounds or whole meal in the modulation of the chronic inflammatory conditions, including intestinal inflammatory diseases [3, 4, 6]. Of these, catechins (e.g., epigallocatechin gallate) and stilbenes (e.g., resveratrol) are some of the most studied grape polyphenols, having well-documented anti-inflammatory and antioxidant properties [6]. For example, (-)-epigallocatechin 3-O-gallate (EGCG) has an anti-inflammatory effect in LPS- induced cancer in the HT-29 colon cell line by decreasing the synthesis of pro-inflammatory mediators (nitric oxide-NO and interleukin -8, IL-8) as well as the activation of inflammatory signalling pathways (mitogen activated kinases, MAPKs and nuclear factor–*k*B, NF-*κ*B). [11]. Also, proanthocyanidin extracted from grape seed had antioxidant effects in rats with 2,4,6-trinitrobenzene sulfonic acid (TNBS)- induced colitis and in dextran sulphate sodium (DSS)-treated mice, increasing the activity of GPx and SOD antioxidant enzymes in serum and colon [12, 13]. Grape waste is also rich in mono- and polyunsaturated fatty acids (PUFA) such as linoleic fatty acid (C18:2, n-6) and oleic fatty acid (C18:1, n-9), also knowing for their beneficial effect on animal and human health. For example, a diet with 4ml/b.w. grape seed oil, containing a high amount of essential PUFAs (linoleic C18:2, n-6, and alpha-linoleic C18:3, n-3 acids) attenuated oxidative stress in hepatic tissues of gamma-irradiated rats, decreasing the expression of inducible nitric oxide synthase (*iNOS*) gene [14]. We also reported previously the positive effects of grape waste in the modulation of intestinal inflammation, in both *in vitro* [15] and *in vivo* [16, 17] studies of IBD (Intestinal Bowel Diseases) using pig as animal models. The *in vitro* studies on Caco-2 cells demonstrated the anti-inflammatory effects of GP which decreased the gene and protein expression level of pro-inflammatory cytokines, signalling molecules MAPKs (p38/JNK/ERK) and nuclear receptor NF-*k*B involved in pro-inflammatory cytokine production [15]. The *in vivo* experimentations on porcine model for human proved the efficacy of GSM (8% into the diet) to restore the epithelial integrity, mucosal barrier functions as well as the innate immunity markers (Toll-like receptors and their associated MyD88/MD2 signalling pathway members) in DSS-challenged piglets [17]. Pig is not only a good model for the study of inflammation in humans, but also one of the farm animals’ species most affected by the inflammation after weaning. The gut and especially the colon are the most vulnerable tissues to inflammation and oxidative stress [18] provoked by weaning changes and pathogens. The entire gut environment is affected, lymph nodes been included [18].

In the present study we evaluated the ability of GSM to counteract oxidative stress in pig by using *in vitro* (porcine cells challenged with *E*. *coli* LPS) and *in vivo* (piglets after weaning challenged with DSS) experimentation. The effect of GSM on several markers involved in oxidant/antioxidant responses (ROS, antioxidant enzymes-SOD, CAT, GPx) and on important components of Keap1/Nrf2 signalling pathway were analysed in IPEC-1 cells, colon and lymph nodes of piglets after weaning.

## 2. Materials and methods

### 2.1. In vitro study

#### 2.1.1 Obtaining of grape seed meal extract

One gram of grape seed meal was mixed with 7 mL (ratio of sample: solvent 1:7 w/v) of 80% acetone and shaken continuously for 24h and then filtered through Whatman filter paper. Samples were concentrated to remove the acetone and the residue was resuspended in water. The aqueous extract was diluted in culture media and used in cell culture experiments.

#### 2.1.2. Determination of polyphenol content and antioxidant activity of GSM extract

The total polyphenols content of GSM extract was determined using Folin-Ciocalteu method, as described by [10]. The results were expressed as mg gallic acid equivalents (GAE)/100 g of GSM. The analyses of individual phenolic compounds (flavonoids and phenolic acids) were carried out by high-performance liquid chromatography (HPLC) on a Jasco Chromatograph (Jasco Corporation, Tokyo, Japan) equipped with UV/Vis detector as described by [19]. The antioxidant activity of GSM extract was evaluated by DPPH (2, 2-diphenyl-1-picryl hydrazyl) and ABTS (2, 2-azinobis (3-ethylbenzothiazoline-6-sulfonic acid)) assays as described by [19]. All tests were performed in triplicate.

#### 2.1.3. IPEC cell culture and treatments

The IPEC-1 cell line (Intestinal Porcine Epithelial Cells) was used as described in [20]. Briefly, the cells were cultured conventionally with 5% foetal bovine serum (FBS1% antibiotic-antimycotic solution (containing 10000 units penicillin, 10 mg streptomycin and 25 μg amphotericin B per ml), 2mM L-Glutamine (Sigma-Aldrich, St. Louis, MO, USA), 1% insulin/transferrin/selenium (ITS) and 5 μg/ml epidermal growth factor (EGF) in Dulbecco’s Modified Eagle Medium DMEM/F-12.

For *in vitro* experiments, cells were seeded on 24-well plates (Nunc, Thermo Fisher Scientific, Waltham, MA, USA) at a concentration of 0.1x10^6^ cells/ml (for qPCR and antioxidant activity), 0.2x10^6^ cells/ml for flow cytometry analysis) until the monolayers were 80% completed. In order to provoke a state of oxidative stress into the cells a challenge with lipopolysaccharide (LPS) from *E*. *coli* was used similarly with our previous study already published, as a model of LPS-induced damage [15]. The concentration of polyphenols in GSM extract used in the present study was chosen based on the results of a MTT cellular viability test which showed that 50μg polyphenols /ml GSM extract did not induce a cytotoxic effect in epithelial intestinal cells, their viability being over 90%. Pure EGCG was used as positive control taken as model the study of Wan et al. who testeddifferent concentrations of EGCG and demonstrated that 50μM of EGCG enhances the epithelial immunological barrier function in IPEC-J2 cells (inducing secretion of certain antimicrobial molecules from the IPEC cells into the culture medium, e.g. pBD-1 and pBD-2) [21].

The cellular treatments were applied as follows:

1. Control: untreated cells;
2. LPS: cell treated with 5μg/ml of LPS () for 24 h; 100 μL culture media was added after 4h;
3. GSM: cells treated (after 4h of incubation) with 100μL of GSM phenolic extract (corresponding to 50μg total polyphenols/mL), for 24h;
4. LPS+GSM: cells treated for 24h with 100μL of GSM phenolic extract (50μg total polyphenols/mL), after 4h of incubation with LPS;
5. EGCG: cells treated (after 4h of incubation) with 100μL (50μM/23μg/ml) of (-)-Epigallocatechin gallate (EGCG, MW 458,37 Da, Sigma–Aldrich, St. Louis, MO, USA), for 24h;
6. LPS+EGCG: cells treated for 24h with 100μL (50μM) of EGCG, after 4h of incubation with LPS.

All solutions used in cell culture were filtered using nonsterile micro-centrifugal filters 0.2μm (Thermo Fisher Scientific, Waltham, MA, USA) and were stored at 4°C.

At the end of incubation time, the cell culture supernatants were collected and stored at -80˚C until the next analyses.

For flow cytometry analyses, after incubation with the treatments, cells were detached with ethylenediaminetetraacetic acid (EDTA)-trypsin, washed with sterile PBS (phosphate buffered saline) and used immediately. For quantitative PCR (qPCR) analysis, the cells were collected, rinsed with sterile PBS, lysed and stored at -80˚C until the next analyses.

#### 2.1.4. Flow cytometry analysis

The quantitative measurement of ROS in cultured IPEC-1 cells was done using Muse® Oxidative Stress Kit (Luminex Corporation, Austin, TX, USA), according to manufacturer’s recommendations. After the cell staining, the relative percentage of ROS positive and ROS negative cells were acquired using Guava® Muse® Cell Analyzer (Luminex Corporation, Austin, TX, USA). Three individual experiment was carried out and ROS analysis was performed in triplicate for each treatment. After the dot plot and histogram analysis, the results were presented as ROS (+) and ROS (-) cell percentages of total cells.

#### 2.1.5. Quantitative PCR analysis

Total RNA extraction and quantitative PCR (qPCR) was performed by using the same methods for both cellular and organs samples.

The extraction of total RNA (tRNA) was performed using specific kit (Qiagen RNeasy mini kit—QIAGEN GmbH, Hilden, Germany), following the instructions of manufacturer. The concentration and quality of isolated tRNAs were evaluated using 2100 Agilent Bioanalyzer (Agilent Technologies, Santa Clara, CA, USA); the complementary DNA (cDNA) was synthetized using the M-MuLV reverse transcriptase kit (Thermo Fischer Scientific, Waltham, MA, USA).

The quantitative PCR (qPCR) was used to evaluate the expressions of genes coding for antioxidant enzymes (catalase—*CAT*, superoxide dismutase—*SOD* and glutathione peroxidase—*GPx*), endothelial nitric oxide synthase (*eNOS*), *iNOS*, *Nrf2* and Nrf2 signalling markers (*Keap1*, *NQO1*, *HO1*). The sequences of primers used are presented in S1 Table. The qPCR reaction set-up and cycling conditions were performed as described by [15]. Two reference genes (*ACTB*: β-actin and *RPL32*: Ribosomal Protein L32) were selected from a panel of six reference genes, using NormFinder Excel-based software [22] and were used for the normalization of qPCR data. The results were analysed using 2^(−ΔΔCT)^ method, and were expressed as relative Fold Change (Fc) compared to untreated (Control) cells. The qPCR analyses were performed in triplicate.

#### 2.1.6. Determination of antioxidant parameters

The activities of CAT and SOD enzymes were measured using Cayman kits (Cayman Chemical, Ann Arbor, MI, USA), according to the manufacturer`s instructions [23]. The Varioskan™ LUX multimode microplate reader (Thermo Fisher Scientific, Waltham, MA, USA) was used for the measurement of the absorbance. The measurements were performed in triplicate.

The total antioxidant capacity (TAC) was determined using a method described by Marin et al. [23]. The results are expressed as μmol TEAC (Trolox equivalent antioxidant capacity)/ml of cell culture supernatant. Three independent replicates were performed for TCA determination.

### 2.2. In vivo study

#### 2.2.1. Animals and experimental diets

Twenty crossbred weaned piglets (TOPIGS-40), 21-days old, were randomly assigned to four experimental groups and fed the following treatments for 30 days: group 1: Control diet (maize-soybean based diet); group 2: control diet challenge with DSS (1 g/b.w./day); group 3: GSM diet (basal diet with 8% grape seed meal included), and group 4: GSM diet challenge with DSS (1 g/b.w./day), as previously described by Pistol et al. [17]. Each experimental group was represented by 5 piglets, with *ad libitum* access to water and feed during the experimental period. The animals were housed in pens (5 piglets/group/pen) within the experimental base of the National Research—Development Institute for Animal Biology and Nutrition, Balotesti, Romania.

*Ethics statements*. The experimental protocol was approved by the Ethical Committee (no. 118/2019) of INCDBNA Balotesti and the animal handling was done in accordance with rules for handling and protection of animals used for experimental purposes with EU Council Directive 98/58/EC and Romanian Law 43/2014 and in compliance with the ARRIVE guidelines.

The chronic inflammation and oxidative stress were induced by two cycles of DSS challenging (days 1–5 and 21–25 of the experiment), consisted of orally administration of 1g/b.w./day of DSS (Dextran sulphate 40 sodium salt, MW = 36–50 kDa, Carl Roth GmbH, Karlsruhe, Germany) to piglets from groups 2 (DSS) and 4 (DSS+GSM) [16, 17]. At the end of the experiment (day 30) pigs (previously starved) were electrically stunned followed by exsanguination (according to the EU Council Directive 2010/63/CE), and samples of the colon and mesenteric lymph nodes were collected on ice and stored at −80˚C until the analyses.

GSM and experimental diets composition including total polyphenol and the different classes of polyphenols as well as polyunsaturated fatty acids (PUFA) and antioxidant capacity (DPPH), was analysed as already described by Pistol et al. [17].

#### 2.2.2. Quantification of ROS in colon and mesenteric lymph nodes

The ROS concentration in colon and mesenteric lymph nodes was quantified using the method described by [24]. Frozen colon and mesenteric lymph nodes samples (100 mg) were homogenized (1:10 w/v) in 1 mL of ice-cold Tris-HCl buffer (40 mM, pH = 7.4), using UltraTurrax homogenizer (IKAWerke GmbH &Co. KG, Staufen, Germany). 100 μl of tissue homogenates were mixed with 1 mL of Tris-HCl buffer and 5 μl of 10 μM DFCA DA (2`,7`-dichlorofluorescein diacetate, Sigma-Aldrich, St. Louis, MO, USA). Another pool of 100 μl of tissue homogenate were mixed with 1 mL of Tris-HCl buffer and used as control of tissue autofluorescence. The samples were incubated for 40 minutes at 37 ˚C, with agitation (Precision Incubators INB 400, Memmert GmbH, Schwabach, Germany). After incubation, 200 μl of each sample was transferred in 96-well optical-bottom plates (Nunc, Thermo Fischer Scientific, Waltham, MA, USA), in duplicate. The ROS analysis was performed in triplicate for each sample. The fluorescence intensity of the samples was measured using Varioskan™ LUX multimode microplate reader (λ_excitation_ = 485 nm and λ_emission_ = 525 nm) (Thermo Fisher Scientific, Waltham, MA, USA).

#### 2.2.3. Determination of antioxidant parameters in colon and mesenteric lymph nodes

The activities of CAT, GPx and SOD enzymes were measured using Cayman kits (Cayman Chemical, Ann Arbor, MI, USA), according to the manufacturer`s instructions [23]. Also, the methods described by Marin et al. [23] were used for evaluation of the TAC and thiobarbituric acid reactive substances (TBARS). The results are expressed as nmol TEAC (Trolox equivalent antioxidant capacity)/g of tissue for TAC and nmol/g sample for TBARS. The analysis of DNA/RNA oxidative damage and of protein carbonyl were performed as described by [25] and carried out in triplicate.

#### 2.2.4. Immnunoblotting analysis of Nrf2 in colon and mesenteric lymph nodes

The Nrf2 protein expression in colon and mesenteric lymph nodes samples was evaluated by Western blotting. Frozen organ samples (100mg) were lysed as described by [16] and the western blotting was performed as described by Marin et al. [23]. Nuclear and cytoplasmic lysates from colon and mesenteric lymph nodes samples were obtained as described by [25]. As primary antibodies rabbit anti-pig Nrf2 and β-actin (both diluted 1:1000 Cell Signaling Technology, Danvers, MA, USA) were used. The horseradish peroxidase-conjugated goat anti-rabbit antibody was used as secondary antibody (1:2000, Cell Signaling Technology, Danvers, MA, USA). For protein detection the Clarity Western ECL Substrate (Bio-Rad Laboratories, Hercules, CA, USA) was used. Micro-Chemi Imager (DNR Bio-Imaging Systems LTD, Israel) and GelQuant software (DNR Bio-Imaging Systems LTD, Israel) were used for immunoblotting images developing and analysis. The results were expressed as the ratio of the expression level of Nrf2 protein to that of β-actin.

### 2.3. Statistical analysis

Results are presented as mean ± SEM. One-way ANOVA tests, followed by Fisher’s procedure of the least square difference were used for the comparison between experimental groups (StatView software 6.0, SAS Institute, Cary, NC, USA), and statistical significance was considered at *p* < 0.05, which are largely detailed in S2 Table. As the sample sizes for both *in vitro* and *in vivo* studies was small, Fisher’s test was used to assure a correct statistical significance. The homogeneity of the results was assessed by Pettitt’s test and Buishand’s test for homogeneity (XLSTAT2020 software). The results were tested for the normal distribution of the data using the NORM.DIST function of MS-Excel. The principal component analysis (PCA) statistical multivariate method and heatmaps (XLSTAT2020 software and ClustVis web tool [26]), were used for cluster analysis of experimental groups, in both *in vitro* and *in vivo* studies.

## 3. Results

### 3.1. Characterisation of GSM extract

Data presented in Table 1 show that the extract used in the *in vitro* studies obtained from GSM by extraction in 80% acetone and resuspended in sterile water had a high content of total polyphenols (3915 mg/100g dry matter). Of these, catechins were the most prevalent polyphenol followed by caffeic acid, epicatechin and rutin. GSM extract showed also a high DPPH capacity.

**Table 1:** Composition and antioxidant activity of GSM extract.

**Flavonoids and phenolic acids (mg/100g dry matter)**	
Gallic Acid	12.50
Catechin	104.70
Vanillic Acid	3.69
Caffeic acid	21.75
Epicatechin	5.09
p-Coumaric Acid	0.36
Ferulic Acid	0.17
Sinapic acid	1.29
Rutin	2.96
**Antioxidant activity (mM trolox)**	
DPPH	43.64
ATBS	3.80
**Total polyphenols (mg/100g)**	3915

### 3.2. In vitro study

The aim of the *in vitro* study was to evaluate the effect of GSM treatment on oxidative stress in IPEC-1 cells

#### 3.2.1. Effect of GSM extract on oxidative stress in LPS-stimulated IPEC-1 cells

**Fig 1:**
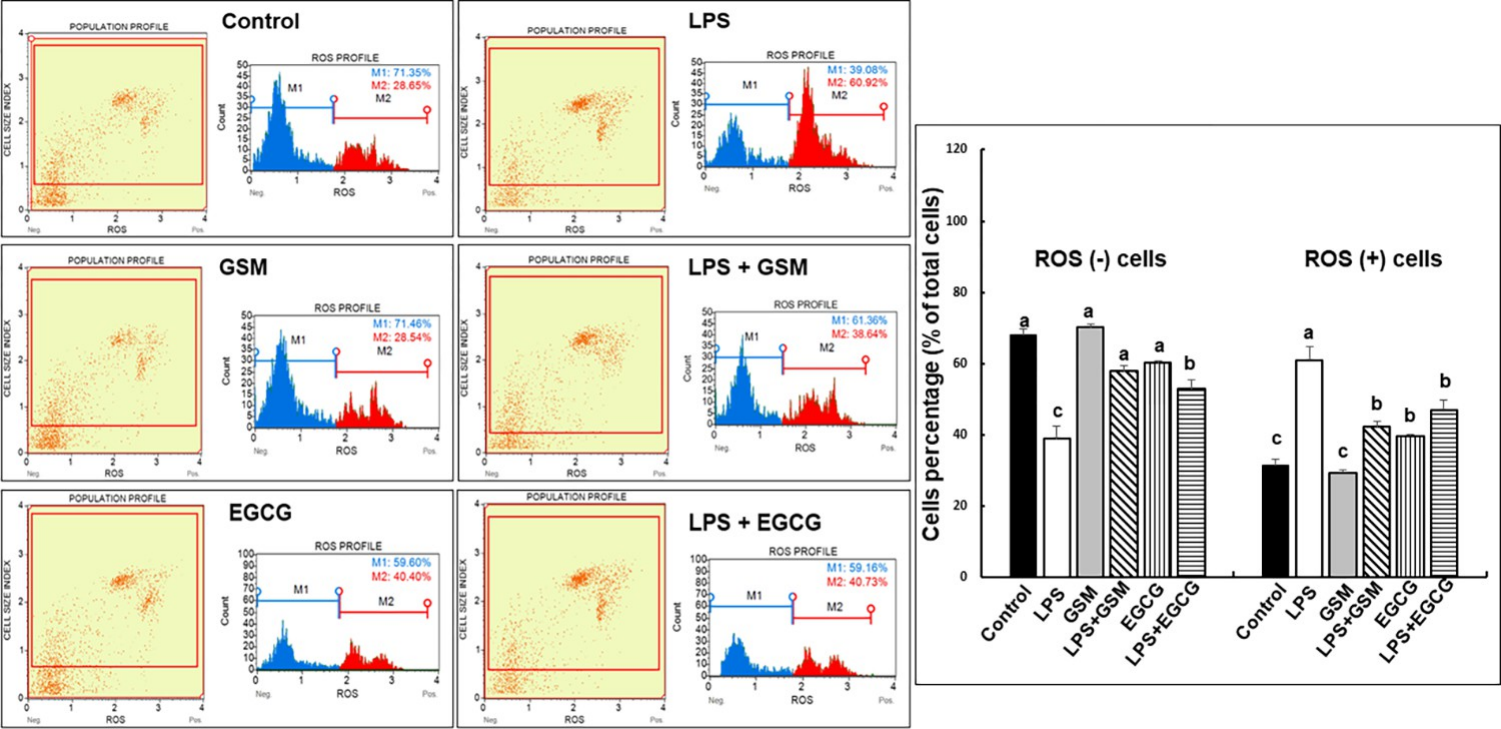
Effects of GSM on the distribution of ROS (+) and ROS (-) cells in IPEC-1 cell population. IPEC-1 cells were incubated with the following treatments for 24 hours: Control = untreated cells; LPS = cells treated with LPS (5μg/ml) + 100 μL culture media, 24h; GSM = cells pre-incubated 4h without LPS and treated after with 100 μL (50μg/mL) of GSM phenolic extract; LPS + GSM = cells pre-incubated with LPS (5μg/ml) 4 h and treated after with 100 μL (50μg/mL) of GSM phenolic extract 24h; EGCG = cells pre-incubated 4h without LPS and treated after with 100 μL (23 μg/ml) EGCG; LPS + EGCG = cells incubated with LPS (5μg/ml) 4 h and treated after with 100 μL (23 μg/ml) EGCG, 24h. Results are presented as means ± standard errors, from three individual experiments. For each treatment, representative dot plots as well as histograms were presented (left panel). ^a, b, c =^ Histograms with unlike superscript letters were significantly different (*p* < 0.050). Three replicates per treatment were analysed, and median values of ROS (+) and ROS (-) cell percentages were showed.

Our results presented in Fig 1 showed that LPS treatment induced an increase of ROS (+) stained cells compared to Control cells (50-times increased, *p* = 0.000018, Fig 1). The addition of both GSM and EGCG treatments reduced the percentages of ROS (+) cells in LPS-treated culture (31- and 23-times decreased, *p* = 0.001 and *p* = 0.043 *vs* LPS, Fig 1).

#### 3.2.2. Effect on antioxidant enzymes genes expression

**Fig 2:**
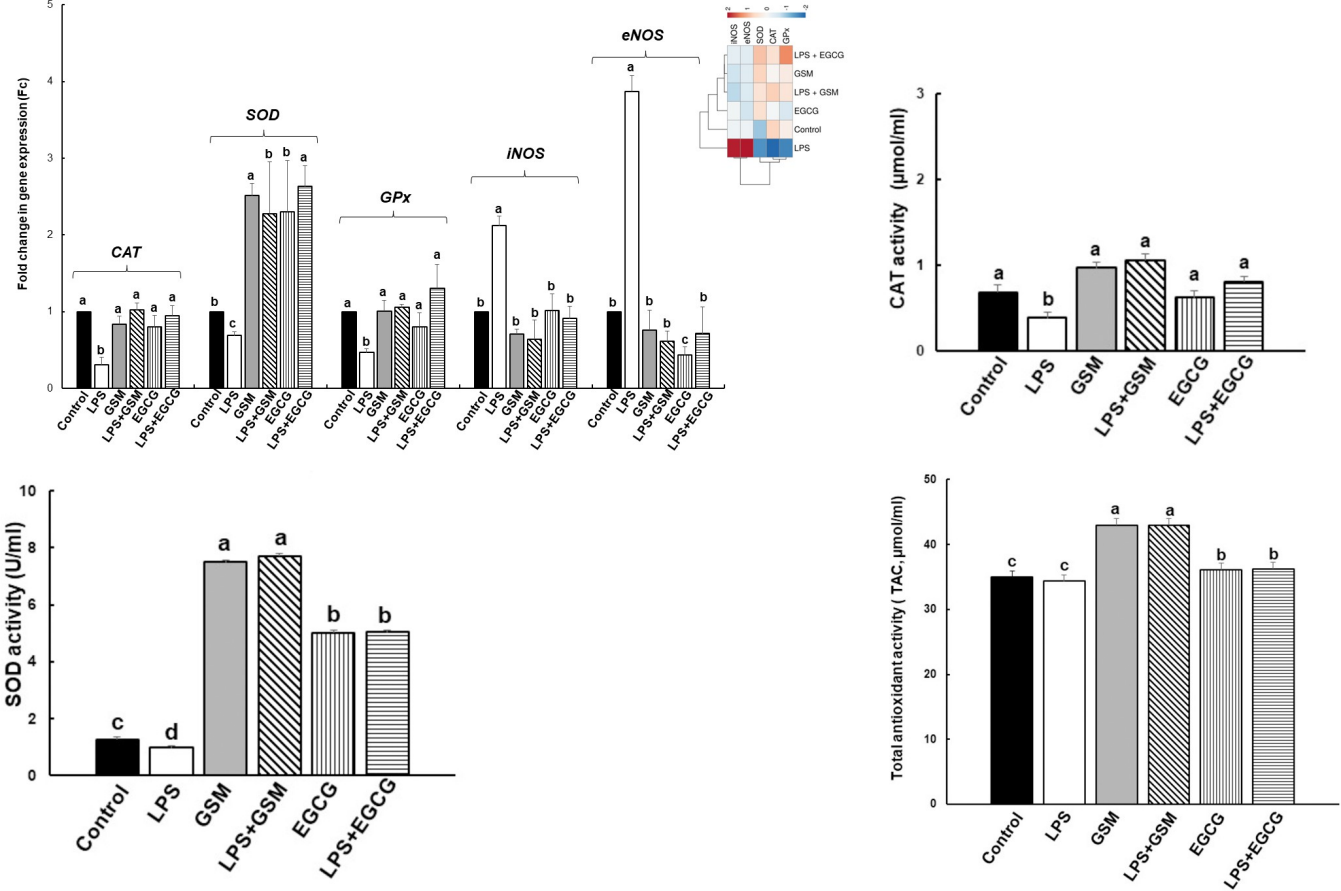
The effects of GSM on the antioxidant genes expression (A) and antioxidant activity (B, C, D) in IPEC-1 cells. IPEC-1 cells were incubated with the following treatments: Control = untreated cells; LPS = cells treated with LPS (5μg/ml) + 100 μL culture media, 24h; GSM = cells pre-incubated 4h without LPS and treated after with 100 μL (50μg/mL) of GSM phenolic extract; LPS + GSM = cells pre-incubated with LPS (5μg/ml) 4 h and treated after with 100 μL (50μg/mL) of GSM phenolic extract 24h; EGCG = cells pre-incubated 4h without LPS and treated after with 100 μL (23μg/ml) EGCG; LPS + EGCG = cells pre-incubated with LPS (5μg/ml) 4 h and treated after with 100 μL (23μg/ml) EGCG, 24h. Results are presented as means ± standard errors, from three experimental series. ^a, b, c =^ Histograms for each group with unlike superscript letters were significantly different (*p* < 0.050). The enzyme activities were expressed as: μmol/ml (CAT), U/ml (SOD), μmol/ml (TAC). The heatmap (the upper right panel) represents antioxidant gene expression levels in experimental groups of cells. The magnitude of gene expression level is represented by a colour scale (top) going from low (blue) to high (red).

To evaluate the effects of GSM extract on the antioxidant response in IPEC-1 cells, the level of genes expression coding for catalase (*CAT*), superoxide dismutase (*SOD*) and glutathione peroxidase (*GPx*), *eNOS* and *iNO*S were analysed by qPCR. LPS treatment (5μg/ml) induced a significant down-regulation of *CAT*, *SOD* and *GPx* gene expression when compared to IPEC-1 unchallenged cells (*CAT*: 0.3 ± 0.1Fc, *p* = 0.018 *vs* Control; *SOD*: 0.6 ± 0.0Fc, *p* = 0.029 *vs* Control; *GPx*: 0.4 ± 0.0Fc, *p* = 0.008 *vs* Control, Fig 2A). The addition of both GSM and EGCG (50μg/ml and 23μg/ml respectively) in LPS treated cells inhibited the LPS action leading to an increase (p<0.05) of *CAT*, *SOD* and *GPx* mRNA level at the Control point (Fig 2A). GSM and EGCG alone had no effect on enzyme activity. By contrast, LPS challenge induced a significant augmentation of *eNOS* and *iNOS* gene expression compared to untreated cells (*eNOS*: 3.8 ± 0.2Fc, *p* = 0.005 *vs* Control; *iNOS*: 2.1 ± 0.1Fc, *p* = 0.012 *vs* Control, Fig 2A). In LPS challenged cells both GSM and EGCG treatments decreased the *eNOS* and *iNOS* gene expression restoring their expression toward the Control level (Fig 2A). When compared to Control, EGCG also down expressed the *eNOS* gene. Heatmap presented in Fig 2A showed that LPS group is clearly different from all other experimental groups, GSM and LPS+GSM groups being the most appropriate to Control samples (Fig 2A).

#### 3.2.3. Effect on the antioxidant enzymes activities

The results of enzymatic activity analysis showed that both CAT and SOD activities were reduced significantly in LPS-stimulated cells compared to untreated control cells (-42% decrease in CAT activity, *p* = 0.050 *vs* Control; -23% decrease in SOD activity, *p* = 0.005 *vs* Control, Fig 2B and 2C) and had no effect on total antioxidant activity (TCA) (Fig 2D). The treatment with GSM (50μg/mL) and EGCG (23μg/ml) in LPS treated cells restored the activity of CAT enzyme toward the Control level. (Fig 2B). We noticed the high antioxidant capacity of EGCG and especially of GSM treatment alone which increase over the Control the level of the three analysed parameters. The strong antioxidant activity of GSM not only annihilated the activity of LPS on SOD activity but also keeps it significantly above the Control (Fig 2C).

### 3.2.4. Effect on the expression of genes coding for signalling markers involved in oxidative stress pathway

**Fig 3:**
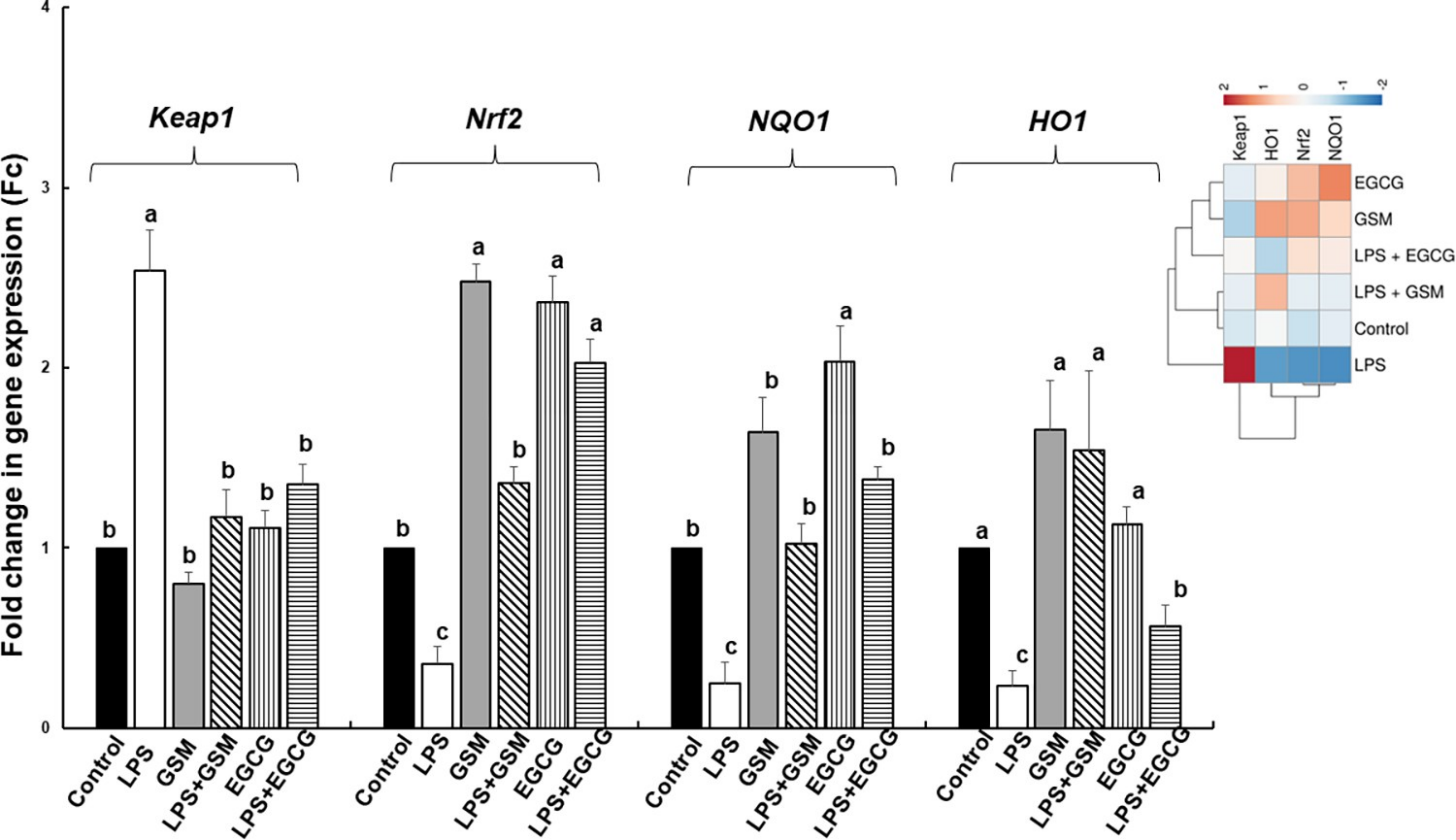
The effects of GSM on the expression of genes coding for Keap1/Nrf2 signalling pathway in IPEC-1 cells. IPEC-1 cells were incubated with the following treatments: Control = untreated cells; LPS = cells treated with LPS (5μg/ml) + 100 μL culture media, 24h; GSM = cells pre-incubated 4h without LPS and treated after with 100 μL (50μg/mL) of GSM phenolic extract; LPS + GSM = cells pre-incubated with LPS (5μg/ml) 4 h and treated after with 100 μL (50μg/mL) of GSM phenolic extract 24h; EGCG = cells pre-incubated 4h without LPS and treated after with 100 μL (23μg/ml) EGCG; LPS + EGCG = cells pre-incubated with LPS (5μg/ml) 4 h and treated after with 100 μL (23μg/ml) EGCG, 24h. Results are presented as means ± standard errors, from three experimental series. ^a, b, c^ Histograms for each group with unlike superscript letters were significantly different (*p* < 0.050). The heatmap (the right panel) represents the *Keap1*, *Nrf2*, *NQO1* and *HO1* gene expression levels of experimental groups. The magnitude of the gene expression level is represented by a colour scale (top) going from low (blue) to high (red).

In our *in vitro* experiment, treatment with 5 μg/ml LPS leaded to a significant increase of *Keap1* gene expression (2.5 ± 0.2 Fc, *p* = 0.021 *vs* Control, Fig 3) and to a reduced expression of *Nrf2*, *NQO1* and *HO1* genes (*Nrf2*: 0.4 ± 0.1Fc, *p* = 0.031 *vs* Control; *NQO1*: 0.2 ± 0.1Fc, *p* = 0.025 *vs* Control; and *HO1*: 0.2 ± 0.1Fc, *p* = 0.013 *vs* Control, Fig 3). The GSM addition to LPS-challenged cells restored the *Keap1* mRNA level to the Control level (Fig 3), and reverted also the inhibitory effects of LPS on *Nrf2*, *NQO1* and *HO1* gene expressions (Fig 3). By contrast, EGCG was less efficacy in counteracting the effect of LPS than GSM for *Nrf2* and *HO1* expression. The antioxidant capacity of the two extracts is again noticeable. The heatmap showed the distinct difference between LPS treated cells and all other experimental treatments (Fig 3).

### 3.3. In vivo study

The aim of the *in vivo* study was to evaluate the effect of GSM dietary inclusion against DSS induced oxidative stress. The capacity of GSM diet to counteract several important markers involved in oxidative stress at local level in colon and mesenteric lymph nodes derived from piglets challenged with DSS was analysed.

#### 3.3.1. Effect on oxidative stress in colon and mesenteric lymph nodes

**Fig 4:**
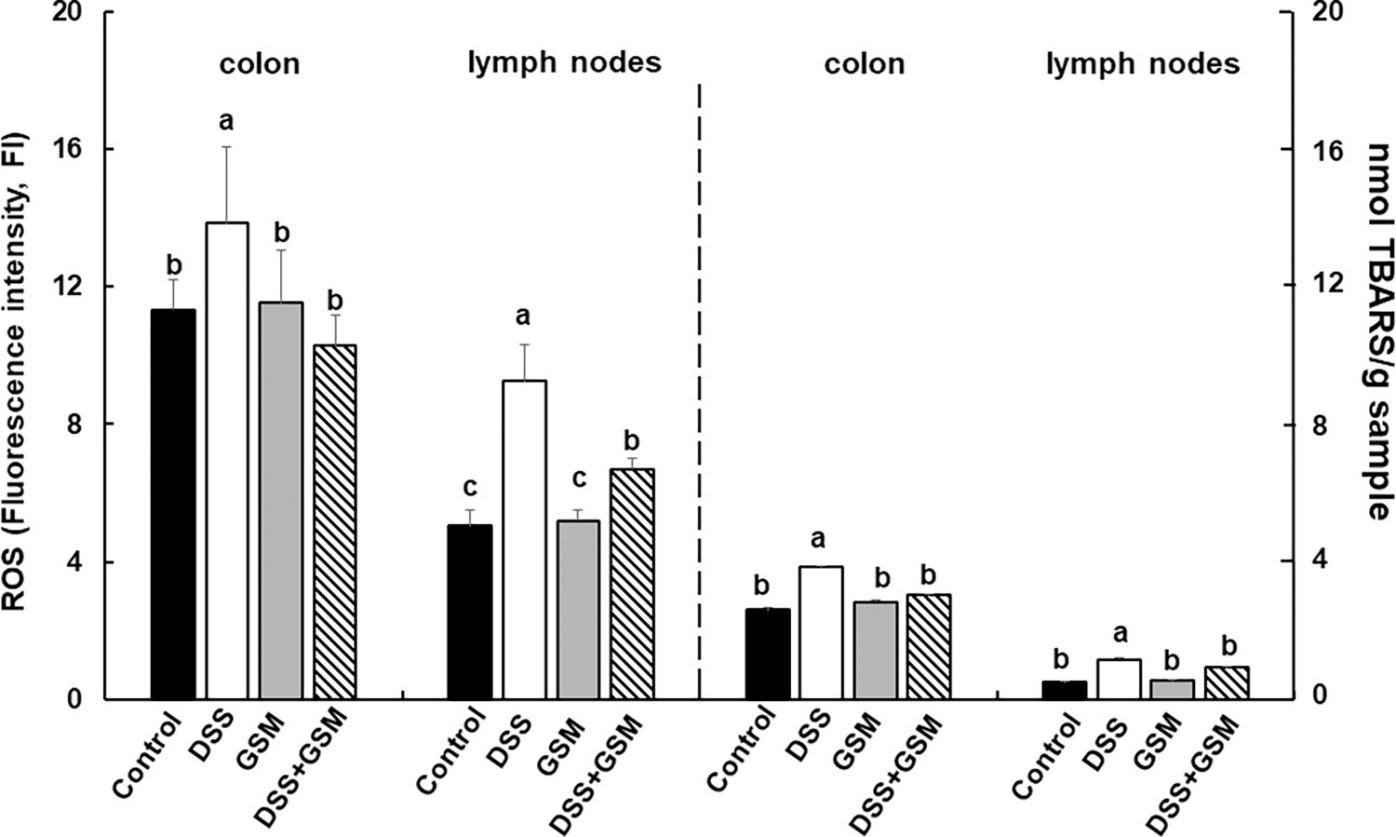
Effect of DSS and GSM diet on ROS and TBARS levels in colon and mesenteric lymph nodes. Unchallenged and DSS-challenged pigs were assigned for 30 days to a control diet (Control and DSS groups) or 8% GSM diet (GSM and DSS + GSM groups). At the end of the experiment, colon and lymph nodes samples from all animals (n = 5) were collected and analysed for ROS and TBARS Results are presented as means ± standard errors. ^a, b, c =^ Histograms for each group with unlike superscript letters were significantly different (*p* < 0.050).

The analysis of ROS showed a significant increase in the colon of DSS-treated piglets (1.5- fold), when compared to Control group, while in colon of piglets treated with DSS and receiving GSM diet (DSS+GSM group), the level of ROS comeback toward the Control (Fig 4). Also, in mesenteric lymph nodes, DSS challenge led to a 2-fold increase of ROS levels when compared to Control (*p* = 0.050, Fig 4). In piglets fed 8% GSM diet the ROS level returned toward the Control, while GSM alone had no effect on ROS neither in colon nor in mesenteric lymph nodes when compared to Control (Fig 4). As marker of lipid peroxidation, TBARS was measured in colon and lymph nodes. The TBARS levels was increased in both organs in DSS-treated piglets (+48%, *p* = 0.0000003 *vs* Control in colon and +127%, *p* = 0.0000002 *vs* Control in mesenteric lymph nodes, Fig 4). 8% GSM inclusion in the diet of DSS-treated piglets reduced TBARS under that of DSS group toward to Control in both colon and mesenteric lymph nodes (Fig 4). GSM treatment alone did not produce any significant effects when compared to Control group.

To evaluate the effects of GSM on protein and DNA oxidation induced by DSS, DNA/RNA oxidative damage and protein carbonyl content were measured. Our results presented in Table 2 showed that DSS induced DNA/RNA oxidative damage by a significant increase in hydroxyguanosine (8-OHG) and 8-hydroxy-2′-deoxyguanosine (8-OHdG) level both in colon (+65% vs Control, *p* = 0.041, Table 2) and in mesenteric lymph nodes (+74%, *p* = 0.017, Table 2). GSM diet reduced the DNA excessive oxidation induced by DSS toward the Control level in both organs (Table 2). Also, protein carbonyl content was increased in colon and mesenteric lymph nodes collected from DSS challenge group (colon: +48%, *p* = 0.045 vs Control; lymph nodes: +30%, *p* = 0.003 vs Control, Table 2). Again, diet with 8% GSM significantly reduced protein carbonyl content when compared to DSS (Table 2). GSM alone had no effect on protein and DNA oxidation in colon and lymph nodes derived from piglets receiving GSM diet (Table 2).

**Table 2:** Effect of GSM diet on DNA oxidative damage and on protein carbonylation.

Parameter	Organ	Experimental treatment *				
		Control	DSS	GSM	DSS + GSM	SEM
DNA oxidative damage (8-oxo dG, pg/mg of tissue)	colon	12,19 ^b^	20,21 ^a^	13,50 ^b^	9,54 ^b^	1,30
	lymph nodes	8,05 ^b^	14,06 ^a^	9,77 ^b^	9,25 ^b^	0,77
Protein carbonyl content (μg/mg of tissue)	colon	0,371^b^	0,550^a^	0,282^b^	0,328^b^	0,031
	lymph nodes	0,136 ^b^	0,177 ^a^	0,104 ^b^	0,116 ^b^	0,008

*Unchallenged and DSS-treated pigs were assigned for 30 days to a control diet (Control and DSS groups) or 8% GSM diet (GSM and DSS + GSM groups). At the end of the experiment, colon and lymph nodes samples from all animals (n = 5) were collected and analysed for DNA oxidation and protein carbonyl content). The results are presented as means.

^a,b^ = values with unlike superscript letters were significantly different (*p* < 0.05).

#### 3.3.2. Effect on genes expression and activity of antioxidant enzymes in colon and mesenteric lymph nodes

**Fig 5:**
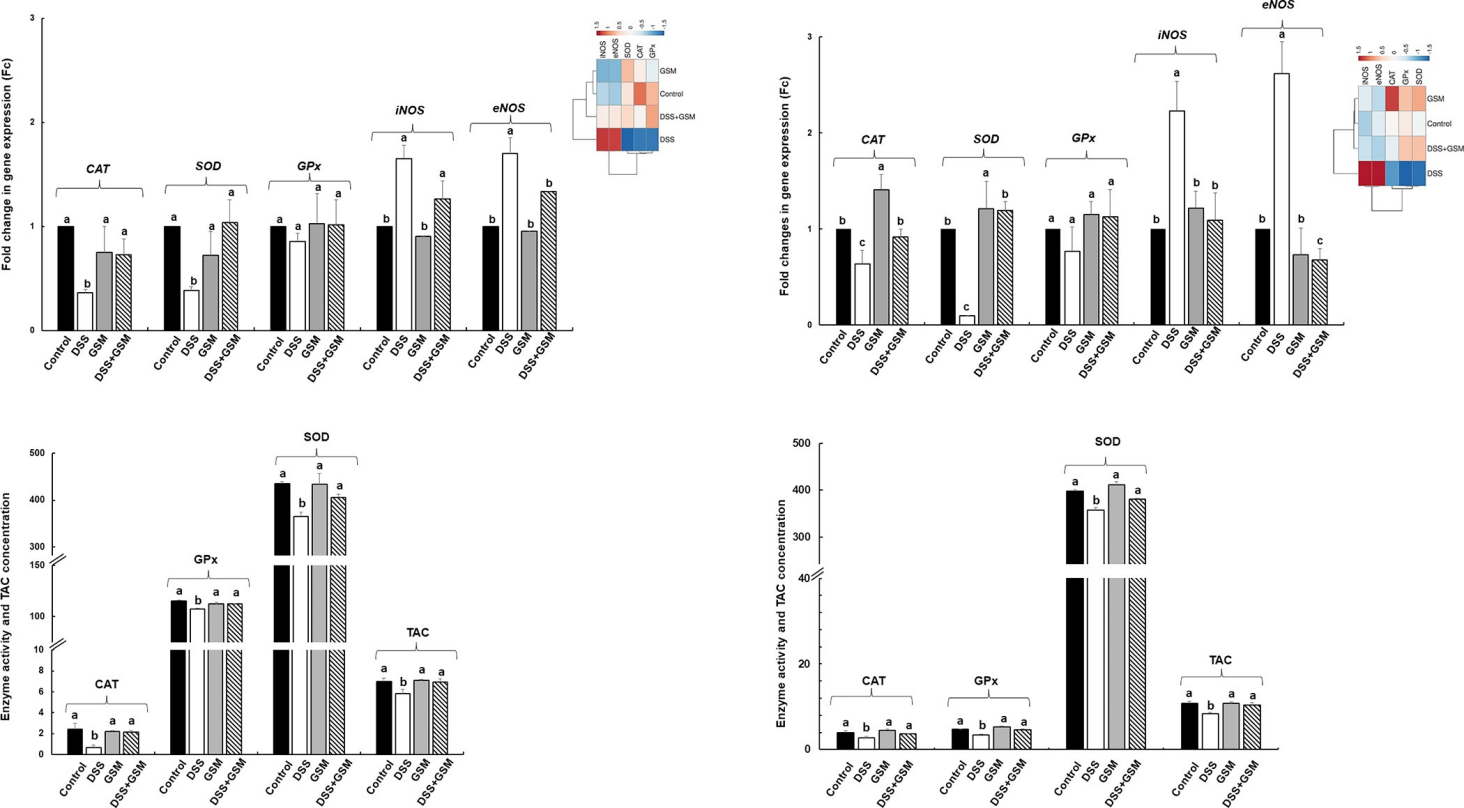
Effect of DSS and dietary GSM treatment on antioxidant gene expression in colon (A), and mesenteric lymph nodes (B) and on enzyme activity and TCA in colon (C) and lymph nodes (D). Unchallenged and DSS-treated pigs were assigned for 30 days to a Control diet (Control and DSS groups) or 8% GSM diet (GSM and DSS + GSM groups). At the end of the experiment, colon and lymph nodes samples from all animals (n = 5) were collected and analysed for gene expression (qPCR), enzymes activity and total antioxidant capacity (TCA). The enzyme activities were expressed as: μmol/min/g tissue (CAT and GPx), U/g tissue (SOD), μmol/g tissue (TAC). Results are presented as means ± standard errors. ^a, b, c =^ Histograms for each group with unlike superscript letters were significantly different (*p* < 0.050). The heatmap (upper right panels) represents gene expression levels in colon (A-right panel) and mesenteric lymph nodes (B–right panel). The blue and red colours correspond to low and high gene expression, respectively.

Similar with *in vitro* results, the qPCR analysis showed that *CAT* and *GPx* gene expressions was down-regulated by DSS treatment, in contrast with *NOS* (*eNOS* and *iNOS*) gene expression significantly up-regulated by DSS-treatment in both colon and mesenteric lymph nodes (Fig 5A and 5B). GSM alone had no effect on *CAT*, *GPx*, *SOD*, *iNOS* and *eNOS* gene expression in colon (Fig 5A), but it increased the *CAT* and *GPx* genes in the mesenteric nodes over the Control values (Fig 5B). The diet with 8% GSM counteracted the effects of DSS treatment, restoring the *CAT*, *GPx*, *iNOS* and *eNOS* antioxidant genes expression toward the Control level, in both colon (Fig 5A) and mesenteric lymph nodes, excepting expression of *iNOS* in lymph nodes which decreased under the Control level (Fig 5B). In both colon and mesenteric lymph nodes collected from GSM and DSS+GSM experimental groups, *SOD* gene expression was unmodified when compared to Control group (Fig 5A and 5B). The heatmap showed also the distinct difference between DSS group and the other experimental groups, in colon (Fig 5A) and mesenteric lymph nodes (Fig 5B), as well the similarity of DSS+GSM group with Control group in colon and of DSS+GSM and GSM groups in mesenteric lymph nodes (Fig 5A and 5B).

The activities of CAT, SOD and GPx enzymes were reduced significantly by DSS compared to Control group, in both colon and mesenteric lymph nodes likewise their gene expression (Fig 5C and 5D). GSM alone had no effect on the antioxidant enzyme activities when compared to Control group (Fig 5C and 5D). By contrast, GSM diet restore the activity of all analysed enzymes in DSS+GSM group at the Control level (Fig 5C and 5D). Similarly, the total antioxidant capacity was significantly reduced in colon and mesenteric lymph nodes collected from DSS-treated group (-16%, *p* = 0.030 *vs* Control and -22% *p* = 0.005 *vs* Control, Fig 5C and 5D.). 8% GSM inclusion in the diet of DSS challenge piglets was able to restore TAC values toward the Control level in their colon and mesenteric lymph nodes (Fig 5C and 5D). GSM treatment alone did not produce any significant effect on TCA when compared to Control group.

#### 3.3.4. Effect on the expression of genes coding for signalling markers involved in oxidative stress pathway

**Fig 6:**
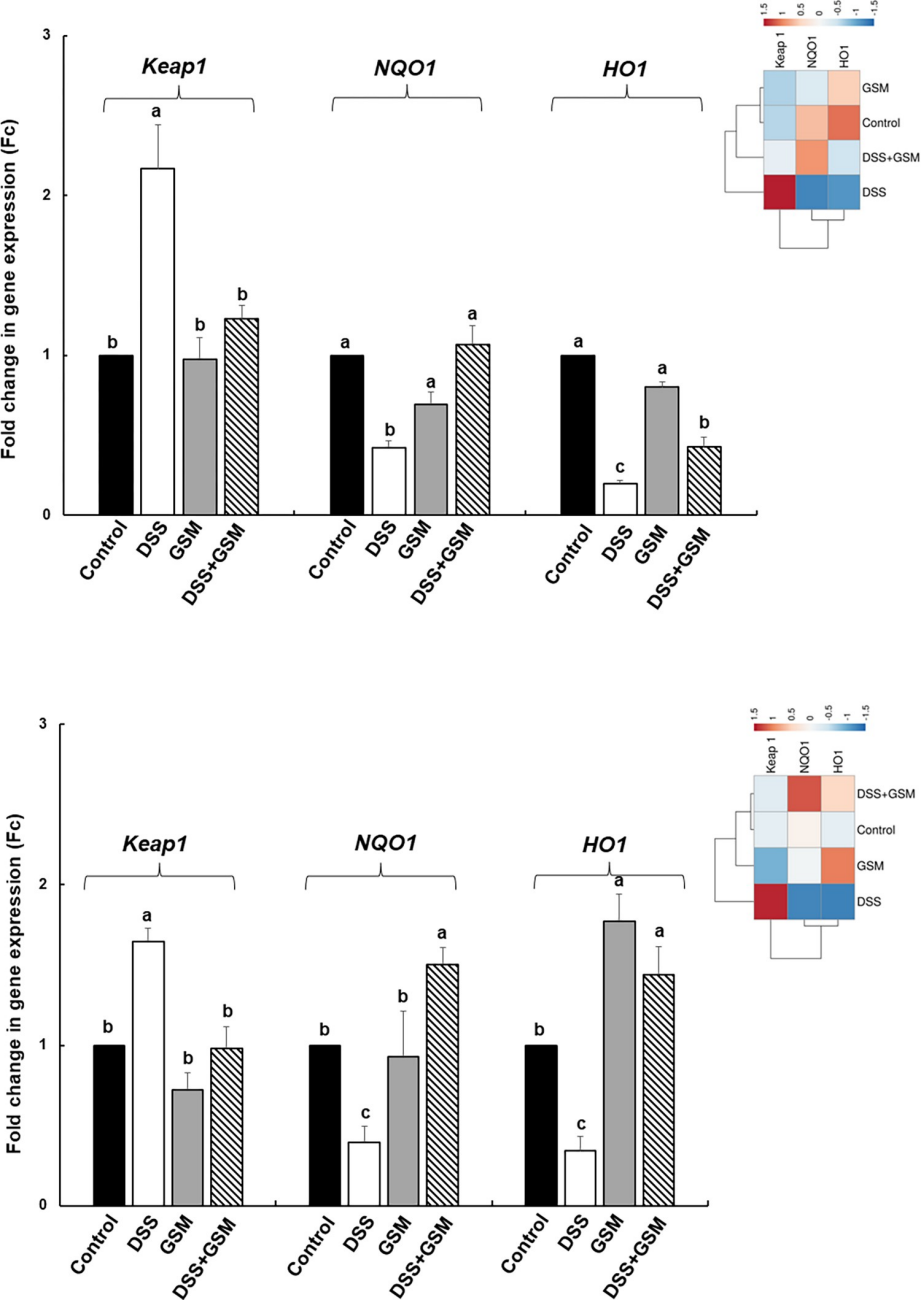
The effect of DSS and dietary GSM treatment on the expression of genes coding for Keap1/Nrf2 signalling pathway in colon (A) and mesenteric lymph nodes (B). Unchallenged and DSS-treated pigs were assigned for 30 days to a Control diet (Control and DSS groups) or 8% GSM diet (GSM and DSS + GSM groups). At the end of the experiment, colon and lymph nodes samples from all animals (n = 5) were collected and analysed for gene expression (qPCR). ^a, b, c =^ Histograms for each group with unlike superscript letters were significantly different (*p* < 0.050). The heatmap (right panels) represents of *Keap1*, *NQO1* and *HO1* mRNA levels in colon (A-right panel) and mesenteric lymph nodes (B–right panel) collected from piglets. The blue and red colours correspond to low and high gene expression, respectively.

To have an overview of antioxidant response mechanism involved by GSM action, we analysed the gene expression of cellular signalling markers such as *Keap1*, *NQO1* and *HO1*, important pathway molecules associated with oxidative stress and inflammation. We found that DSS challenge induced an up-regulation of *Keap1* gene and a down-regulation of gene expression coding for *NQO1* and *HO1*, downstream targets of Nrf2 signalling pathway (- 57%, *p* = 0.0010 *vs* Control and -80%, *p* = 0.002 *vs* Control, respectively, Fig 6A) (+116%, *p* = 0.043 *vs* Control, Fig 6A) in colon. Similarly, *Keap1* expression increased significantly (+65%, *p* = 0.004 *vs* Control, Fig 6B) and *NQO1* and *HO1* decreased also in significant manner (- 60%, *p* = 0.005 *vs* Control and -65%, *p* = 0.007 *vs* Control, Fig 6B) in mesenteric lymph nodes collected from DSS-challenged piglets. The diet including 8% GSM restored *Keap1* and *NQO1* signalling genes expression affected by DSS to the Control level in colon and only of *Keap1* in lymph nodes, but it was not able to restore *NQO1* in lymph node (Fig 6A and 6B). Also, GSM diet was not able to return the *HO1* expression at Control level in colon and in lymph nodes of piglets challenged with DSS (Fig 6A and 6B). GSM alone had no effect in colon but increased *HO1* expression in lymph nodes (*p*<0.05).

The differences between the experimental treatments are shown also in heatmaps presented in Fig 6A and 6B. DSS group have a specific pattern, which clearly differentiated this group from the rest of experimental groups.

**Fig 7:**
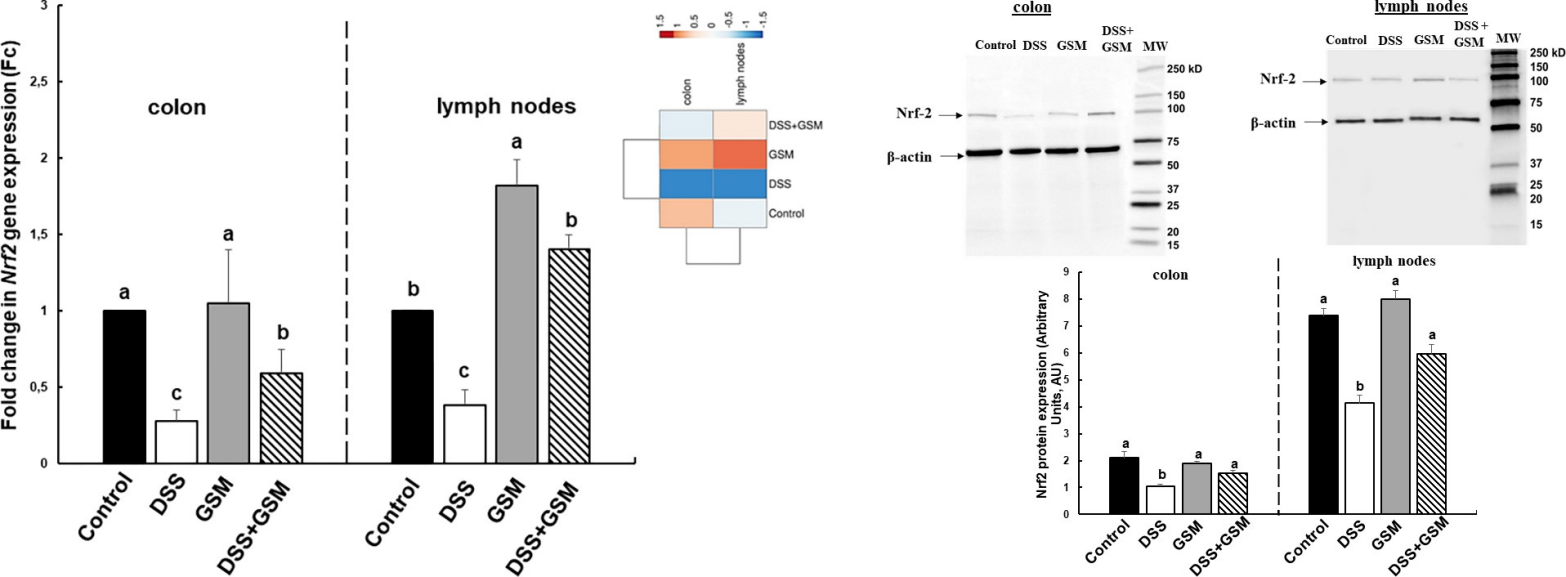
Expression of Nrf2 gene (A) and protein (B) in colon and mesenteric lymph nodes under DSS and GSM action. Unchallenged and DSS-treated pigs were assigned for 30 days to a Control diet (Control and DSS groups) or 8% GSM diet (GSM and DSS + GSM groups). At the end of the experiment, colon and mesenteric lymph nodes samples from all animals (n = 5) were collected. (A) The *Nrf2* gene expression was analysed by qPCR and expressed as Fc. Results are presented as means ± standard errors. (B). The Nrf2 protein expression levels were expressed as arbitrary units (A.U.) as means ± standard errors of the mean (SEM). ^a, b, c^ Histograms for each group with unlike superscript letters were significantly different (*p* < 0.050).

Both gene and protein expression of the Nrf2 signalling marker were analysed in colon and mesenteric lymph nodes. A significant down-regulation of *Nrf2* mRNA was found in samples collected from DSS-challenged animals compared to Control piglets (colon: -72%, *p* = 0.044 *vs* Control; mesenteric lymph nodes: -62%, *p* = 0.049 *vs* Control, Fig 7A.) and a similar effect was observed when we analysed the Nrf2 protein expression. DSS treatment induced a reduction of Nrf2 protein level in DSS-group (-49%, *p* = 0.020 *vs* Control in colon; -44%, *p* = 0.0004 *vs* Control in mesenteric lymph nodes, Fig 7B).

**Fig 8:**
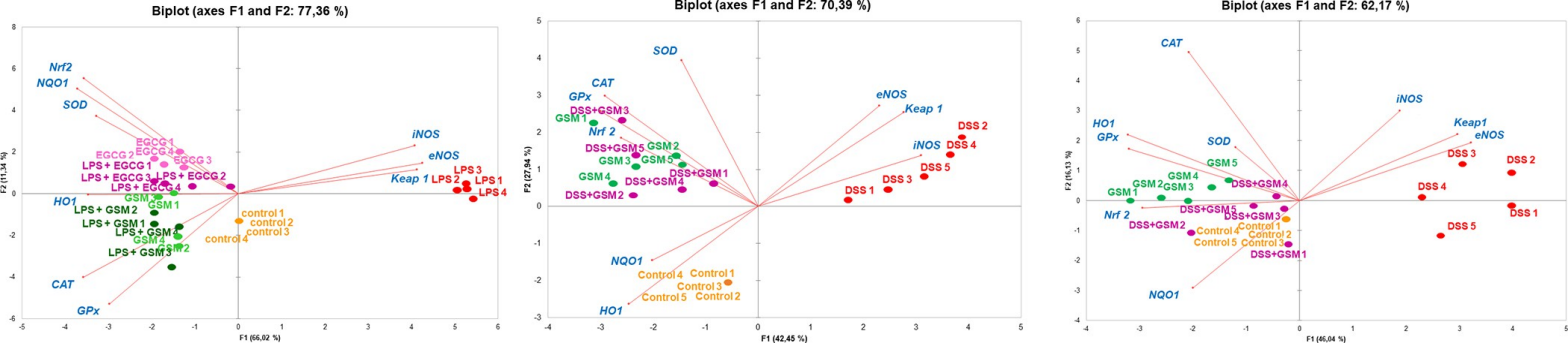
Principal component plots experimental groups with antioxidant genes based on qPCR data set in IPEC-1 cells (A), colon (B) and mesenteric lymph nodes (C). The loading plot with differentially expressed genes in relation to the largest portion of the variance among experimental groups.

In DSS+GSM group, Nrf2 gene and protein expression was restored in lymph nodes toward the Control in response to dietary GSM, also in colon for protein expression, but not for its gene which remained under Control (Fig 7A and 7B). GSM alone in the diet increased significantly (+82%, *p* = 0.036 *vs* Control) the *Nrf2* gene, but not the protein expression in lymph nodes compared to Control and had no effect on Nrf2 (gene or protein expression) in colon (Fig 7A and 7B). Heatmap presented in Fig 8A showed the differences between the DSS group and the other experimental groups in colon and mesenteric lymph nodes (Fig 7A).

To evaluate the effects of DSS and experimental diets on the nuclear translocation of Nrf2 in the nucleus, the expression of Nrf2 was analysed by western blot in cytoplasmic and nuclear extracts from colon and mesenteric lymph nodes. The results presented in Table 3 showed that DSS treatment induced a sequestration of Nrf2 protein in cytoplasm, the level on Nrf2 expression being increased in cytoplasmic extracts collected from both colon and lymph nodes samples of DSS-treated piglets compared to Control group (Table 3, 56.88% *vs* 42.92%, *p* = 0.012 in colon samples and 52.32% *vs* 45.58%, *p* = 0.013 in lymph nodes). The inclusion of GSM in the diet restored the levels of cytoplasmic and nuclear expression of Nrf2 to the Control level (Table 3). GSM alone had no effect on the expression of Nrf2 neither in cytoplasmic nor in nuclear extracts (Table 3).

**Table 3:** Expression of Nrf2 protein in cytoplasmic and nuclear lysates of colon and mesenteric lymph nodes under DSS and GSM action.

Nrf2 protein expression (% of total Nrf2 protein expression)		Experimental treatment *			
		Control	DSS	GSM	DSS + GSM
colon	cytoplasmic Nrf2	42.92%^b^	56.88%^a^	44.15% ^b^	47.44% ^b^
	nuclear Nrf2	57.08% ^a^	43.12% ^b^	55.85% ^a^	52.56% ^a^
lymph nodes	cytoplasmic Nrf2	45.58% ^b^	52.32% ^a^	49.99% ^b^	48.59% ^b^
	nuclear Nrf2	54.42% ^a^	47.68% ^b^	50.01% ^a^	51.41% ^a^

*Unchallenged and DSS-treated pigs were assigned for 30 days to a Control diet (Control and DSS groups) or 8% GSM diet (GSM and DSS + GSM groups). At the end of the experiment, colon and mesenteric lymph nodes samples from all animals (n = 5) were collected. The Nrf2 protein expression levels were expressed as arbitrary units (A.U.) and presented as percentages from total Nrf2 expression in each sample.

^a, b^ Histograms for each group with unlike superscript letters were significantly different (*p* < 0.050).

#### 3.3.5. Principal component analysis

To evaluate the correlation between gene expression resulted from *in vitro* and *in vivo* experimental treatments, the qPCR data set were subjected to PCA analysis. For *in vitro* study, the PCA presented in Fig 8A showed that LPS-treated cells form a distinct separate cluster from the other treated cells (red cluster). Interestingly, the LPS+GSM and GSM clusters are closely related and close as well to the distinct cluster of Control (green and light green clusters); the same similarity was observed for the LPS+EGCG and EGCG clusters (purple and pink clusters). LPS treated IPEC-1 cells was associated with a high expression of *eNOS*, *iNOS* and *Keap1* genes (Fig 8A). The PCA analysis showed an overlap of genes overexpressed in EGCG-treated cell groups. The high levels of *CAT*, *GPx* and *HO1* mRNAs were related to the GSM and LPS + GSM groups, and those of *SOD*, *Nrf2* and its substrate *NQO1* mRNAs were associated with EGCG and LPS + EGCG group (Fig 8A).

Similar results were obtained in the *in vivo* experiment. In colon (Fig 8B) as well as in mesenteric lymph nodes (Fig 8C) the DSS group is clearly separated in the first principal component, being distinctly from Control, GSM and DSS+GSM groups. The DSS+GSM cluster is closely positioned to GSM group in colon (Fig 8B) and DSS+GSM, GSM and Control clusters are closely in mesenteric lymph nodes (Fig 8C). In the two organs analysed the DSS group was associated with a high expression of *iNOS*, *eNOS* and *Keap1* genes (Fig 8B and 8C). In colon, the high level of *CAT*, *GPx*, *SOD* and *Nrf2* mRNAs was related to GSM and DSS+GSM groups, and *NQO1* and *HO1* expressions were associated with Control group (Fig 8B). In mesenteric lymph nodes, the GSM group was associated with the increased expression of *CAT*, *GPx*, *SOD* and *HO1* genes, while high levels of *Nrf2* and *NQO1* mRNAs were related to Control group (Fig 8C). The analysis of DSS+GSM group showed an overlap with genes overexpressed in both Control and GSM groups (Fig 8C).

## 4. Discussions

The production of ROS is a key event in intestinal inflammation, the uncontrolled oxidative stress with overproduction of ROS exceeds the defence mechanisms causing epithelial integrity and barrier function impairment and also an inefficient immune response [1, 27, 28]. Indeed, our previous studies demonstrated that the intestinal DSS-induced inflammation resulted in the downregulation of tight junction proteins expression and of their regulators and an increase in pro-inflammatory cytokine production [16, 17]. In the present study we demonstrated that bioactive compounds from grape seed meal are able to reduce the ROS production. Certainly, the addition of GSM extract in IPEC-1 cell cultures reduced the level of ROS (+) cells after LPS treatment (Fig 1). These results were in agreement with a previous study of Kovacs et al., which demonstrated that the treatment of IPEC-J2 cells with grape seed oligomeric proanthocyanidin (GSOP) extract reduced the ROS levels in LPS-treated cells [29]. Similarly, procyanidin A2, a polyphenolic compound isolated from grape seeds, showed an inhibitory effect on an LPS-induced ROS production in RAW264.7 macrophages [30]. In the study herein the synthetic polyphenol EGCG used as positive control diminished the percentages of ROS (+) cells suggesting that the reducing effect of GSM extract is due to its higher content in polyphenols (5567.22 mg GAE/100 g total polyphenols/sample, 48.93 mg/100 g catechin and, 48.23 mg/100 g epicatechin/sample) [16], known for their antioxidant potential. This property was also manifested on *CAT*, *SOD* and *GPx*, three components of the basic antioxidant defence system against ROS imbalance [28]. In our study, GSM extract was able to restore toward the Control level the gene expression of *CAT*, *SOD* and *GPx* genes down-regulated by LPS challenge (Fig 2A) and to increase CAT and SOD enzymatic activity and total antioxidant capacity in IPEC-1 cells over that of Control and LPS and even of that produced by EGCG polyphenol (Fig 2B).

It seems that the active compounds from grape seed exert their beneficial action irrespective of the type of cells and of animal species on which its act. In ruminants, results obtained by Yang et al., demonstrated that bioactive compounds from grape seed extract (GSE, 10 μg/ml) improved the gene expression and activity of CAT and SOD enzymes in muscle cells isolated from 3-week-old goat stressed with H_2_O_2_ (100 μM) [31]. The results highlighted a bidirectional modulatory effect of GSE because in the unstressed cells the dose of 10 μg/ml of GSE attenuated the gene expression as well as the enzyme activity of CAT and SOD. This shows that special attention should be paid to the use of these compounds which have to be correlated with the state of animal health. In contrast with our results, GSE increased GPx activity in both untreated or treated primary muscle cells. In human Caco-2 cells, apple proanthocyanidin proved also protective effects against LPS-induced oxidative stress, including the increase of *SOD*, *CAT*, and *GSH-Px* mRNA expression [32]. The anthocyanin malvidin (50, 100 and 200 μM), which is the most abundant polyphenol in red wine, reduced the LPS-induced oxidative stress in human THP-1 monocytic cells by increasing the activities of SOD and GPx antioxidant enzymes [33]. Also, different concentrations of malvidin and resveratrol (5, 10, 20, 50 μM) attenuated ROS production in RAW 264.7 murine macrophages challenged with LPS [34].

In order to have a complete picture of GSM effects and taken into consideration the simpleness of the *in vitro* system, *in vitro* studies were followed by an *in vivo* experimentation. Our results obtained in the *in vitro* were confirmed by the results from the *in vivo* trial. Indeed, the diet including 8% GSM restored the *CAT* and *GPx* antioxidant gene expression and their corresponding activities toward to Control level, both in the colon and mesenteric lymph nodes of piglets challenged by DSS (Fig 5A–5D). Using the same percentage of dietary GSM inclusion (8%), Taranu et al. [35] reported that 8% GSM leads to the recovery of the activity of CAT, SOD and GPx enzymes in colon of piglets fed a diet contaminated with aflatoxin B1, one of the most potent mycotoxins, known for the oxidative stress induction. Studies from the literature have shown that the efficacy of bioactive compounds derived from grape by-products in supporting certain components of the antioxidant system during weaning period is manifested even at lower concentrations. For example, the *in vivo* studies of Hao et al. [36] and Chedea et al. [10] revealed that piglets fed 50 mg grape seed procyanidins /kg feed or a diet included 5% grape pomace leaded to an increase in SOD, CAT and GPx activity and in the total antioxidant capacity with a reduction of lipid peroxidation in duodenum and colon. By contrast, Gessner et al. found a decreased in gene expression of *SOD1*, *GPx1* and *Nrf2* in the duodenal mucosa and plasma of piglets receiving 1% grape seed and grape marc meal feed additive [37].

The ROS-generating reactions are catalysed by different enzymes, such as nitric oxide synthases (NOS). The endothelial NOS (eNOS) and inducible NOS (iNOS) are different isoforms of NOS, their increased levels being associated with decreased antioxidant activity [38]. In our study, both *in vitro* and *in vivo* addition of GSM restored the *eNOS* and *iNOS* antioxidant genes expression toward the Control level in LPS-treated IPEC-1 cells and in colon and mesenteric lymph nodes derived from DSS-treated piglets, implying once again the capacity of GSM to ameliorate the ROS-induced damage at intestinal level (Figs 2A, 5A and 5B). Accordingly, other authors observed a similar effect of different by-product grape compounds in *in vitro* on cells or *in vivo* in pig. Thus, the protein expression of iNOS was attenuated in RAW264.7 macrophages under the action of proanthocyanidins A1 and A2 isolated from grape seeds [30, 39]. 5% inclusion of grape seed cake in the diet of finishing pigs resulted in a decrease of *eNOS* and *iNOS* gene expression in liver [40] while 10 mg/kg body weight or 20mg/kg feed of resveratrol, a naturally occurring polyphenol widely present in grapes and red wine attenuated the DSS-induced upregulation of iNOS protein expression in colon of mice [41, 42]. Review paper of Gessner et al. [43] on this subject have shown that grape polyphenols are capable of two important actions that can prevent and stop the development of oxidative stress: the direct cleansing of ROS and secondly, the activation of Nrf2 signalling pathway. The direct antioxidant action occurs in the gut where the concentration of polyphenols is very high and where they are directly exposed to the intestinal epithelium [43].

Nrf2, a member of the leucine regulatory protein family, is a critical transcription factor in the regulation of oxidative stress [3] and for the maintaining of cell homeostasis. Under normal conditions, the upstream repressor of Nrf2, Kelch-like epichlorohydrin-related protein-1 (Keap1) binds to Nrf2, fixed it in the cytoplasm and is responsible for the Nrf2 proteasome degradation and the maintaining Nrf2 at a low level [44, 45]. Under activation conditions, Nrf2 is released from the complex, enters and accumulate in the nucleus where recognize and activate the antioxidant response element (ARE) leading further to the induction of so-called vitagenes encoding for antioxidant enzymes, *SOD*, *GPx*, *CAT* and also for *NQO1* (NAD(P)H dehydrogenase [quinone] 1) and *HO1* (hemoxygenase 1) [46]. Our study demonstrated that GSM extract or dietary GSM restored the LPS and DSS-supressed Nrf2 gene and protein expression decreasing also the *Keap1* mRNA toward the Control level (Figs 3, 8A and 8B) in IPEC1 cells and in the key organs (colon and mesenteric lymph nodes) of DSS-challenged piglets which open the activation of Nrf2 signalling pathway followed by the activation of the antioxidant defence system. Indeed, in cells and piglet’s tissue the *Nrf2* and its downstream *NQO1* and *HO1* recover the Control level under GSM action (Figs 3, 8A and 8B). In the same way, Wang et al., demonstrated that the *in vitro* treatment of RAW264.7 cells with proanthocyanidin A2 (20, 40, 80 μM) up-regulated gene and protein expression of Nrf2 and HO1 and reduced Keap-1 protein expression [30] and are responsible for the Nrf2 translocation from the cytoplasm into the nucleus. Also, Fan et al. reported that the anthocyanin malvidin (5, 10 and 20 mg/kg of body weight) decreased the cytoplasmic expression of Nrf-2, alleviating the LPS-induced oxidative stress via up-regulating the Nrf2 signalling pathway in mice [47]. By contrast, in another type of cells, Caco-2/15 intestinal cells iron/ascorbate (Fe/Asc), proanthocyanidins (250 μg/mL) had no effect on Nrf2 protein expression, but down-regulated the Keap1 expression. Also, a concentration of 4 μg/ml of grape seed extract did not significantly affect the Nrf2 substrate *HO1* gene expression [48] in H_2_O_2_- treated IPEC-J2 cells. Not only *in vitro* but also *in vivo* studies demonstrated the ability of polyphenols from different sources to modulate the Keap1/Nrf2 pathway. For example, polyphenolic extract of green pea hull can activate Keap1/Nrf2/ARE signalling pathway, by reducing the Keap1 protein expression and up-regulating the Nrf2 protein and *NQO1* and *HO1* genes in colon derived from DSS-treated mice [49]. Similar results were reported by Sun et al. [50], which showed that quercetin polyphenol (200 mg/kg of diet) attenuated the LPS-induced inhibition of Nrf2 activation and downstream gene expression, including *HO1* and *NQO1* in broiler chickens.

**Fig 9:**
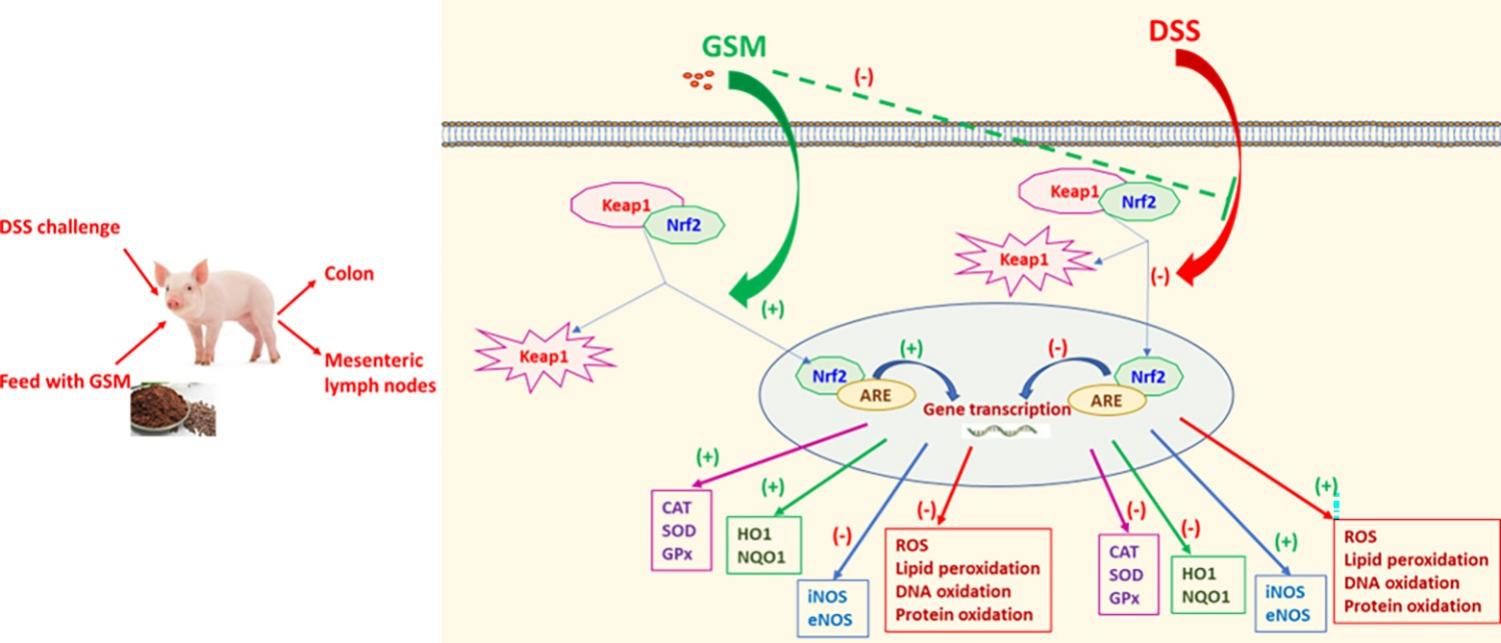
The postulated mechanism of action of DSS and GSM on oxidative stress markers and signalling. DSS treatment reduced the nuclear translocation of nNrf2 into the nucleus leading to (i) stimulation of ROS production and the oxidative degradation of DNA, proteins and lipids; (ii) reduction of the gene expression and activity of antioxidant enzymes, CAT, SOD, GPx and increase of eNOS and iNOS expression. GSM showed anti-oxidant properties counteracting the pro-oxidant response induced by DSS and restoring the levels of endogenous antioxidant enzymes in colon and mesenteric lymph nodes. These effects were mediated via Nrf2 signalling pathway by restoring the expression of *Nrf2*, *Keap1*, *HO1* and *NQO1* genes.

Considering these results, we hypothesized that the key mechanism by which grape waste rich polyphenols and other bioactive compounds exert a beneficial effect on oxidative stress in pigs is the activation of Nrf2 signalling pathway (Fig 9). It has been considered that the antioxidant effect is pronounced in the gut because in the intestinal lumen the concentration of polyphenols is very high. Also, they are directly exposed to the intestinal epithelium [43].

## 5. Conclusions

In conclusion, our results indicated that grape seed meal had the potential to attenuate the oxidative stress associated to inflammation occurred very often in pigs after weaning. The diet including 8% GSM reduced ROS production after DSS treatment and restored the gene expression and activity of antioxidant enzymes, CAT, SOD, GPx, eNOS and iNOS as key components of the defence system at intestinal level. When acting alone GSM led to a significant induction of total antioxidant capacity and enzymes activity in colon and lymph nodes, vulnerable to oxidative reactions. It might be a promising feed alternative in nutritional strategy for piglets to maintain animal health. Similar results were obtained in the *in vitro* LPS-challenged IPEC-1 cells. These effects were mediated via Nrf2 signalling pathway in both *in vitro* and *in vivo* studies.

## Supporting information

S1 Table The sequences of primers used for qPCR amplification. (DOCX)

S2 Table p-values for the parameters analysed in the in vitro and in vivo experiments. (DOCX)

S1 Raw images (PDF)
