# Supplementary material for: Grape seed meal by-product is able to counteract oxidative stress induced by lipopolysaccharide and dextran sulphate in IPEC cells and piglets after weaning
Source: PLoS One. 2023 Apr 13;18(4):e0283607. doi: 10.1371/journal.pone.0283607 (PMC10101422; doi:10.1371/journal.pone.0283607)
Supplement: S1 Table — (DOCX) [file pone.0283607.s001.docx]

**Supplementary File S1. Table S1. The sequences of primers used for qPCR amplification.**

| **Gene** | **Description** | **Accession no.** | **Primer source** | **Primer sequence (5`→3`)** | **Orientation** | **Amplicon length**  **(bp)** | **References** |
| --- | --- | --- | --- | --- | --- | --- | --- |
| *CAT* | Catalase | NM_214301.2 | Pig | CTTGGAACATTGTACCCGCT | forward | 241 | [1] |
|  |  |  |  | GTCCAGAAGAGCCTGAATGC | reverse |  |  |
| *SOD* | Superoxide dismutase | NM_001190422.1 | Pig | GAGACCTGGGCAATGTGACT | forward | 139 | [1] |
|  |  |  |  | CTGCCCAAGTCATCTGGTTT | reverse |  |  |
| *GPx* | Glutathione peroxidase | NM_214201.1 | Pig | GGAGATCCTGAATTGCCTCAAG | forward | 62 | [1] |
|  |  |  |  | GCATGAAGTTGGGCTCGAA | reverse |  |  |
| *eNOS* | Endothelial Nitric Oxide Synthase | NM_214295.1 | Pig | CCCTACAACGGCTCCCCTC | forward | 129 | [2] |
|  |  |  |  | GCTGTCTGTGTTACTGGATTCCTT | reverse |  |  |
| *iNOS* | Inducible Nitric Oxide Synthase | NM_001143690.1 | Pig | GGAGCCATCATGAACCCCAA | forward | 73 | [2] |
|  |  |  |  | GTAGAAGCTCGTCTGGTGGG | reverse |  |  |
| *Nrf-2* | Nuclear factor erythroid 2–related factor 2 | XM_005671982.1 | Pig | CCCATTCACAAAAGACAAACATTC | forward | 72 | [1] |
|  |  |  |  | GCTTTTGCCCTTAGCTCATCTC | reverse |  |  |
| *Keap-1* | Kelch-like ECH-associated protein 1 | XM_021076667.1 | Pig | ACGACGTGGAGACAGAAACGT | forward | 56 | [3] |
|  |  |  |  | GCTTCGCCGATGCTTCA | reverse |  |  |
| *NQO1* | NAD(P)H:quinone acceptor oxidoreductase 1 (NQO1) | NM_001159613.1 | Pig | GTATCCTGCCGAGACTGCTC | forward | 134 | [4] |
|  |  |  |  | TAGCAGGGACTCCAAACCAC | reverse |  |  |
| *HO1* | Heme oxygenase-1 | NM_001004027.1 | Pig | ATGTGAATGCAACCCTGTGA | forward | 89 | [5] |
|  |  |  |  | GGAAGCCAGTCAAGAGACCA | reverse |  |  |
| *B-2 mg* | β 2 microglobulin | NM_213978 | Pig | TTCTACCTTCTGGTCCACACTGA | Forward | 162 | [1] |
|  |  |  |  | TCATCCAACCCAGATGCA | Reverse |  |  |
| *GAPDH* | Glyceraldehyde-3-Phosphate Dehydrogenase | NM_001206359.1 | Pig | ACTCACTCTTCTACCTTTGATGCT | Forward | 100 | [1] |
|  |  |  |  | TGTTGCTGTAGCCAAATTCA | Reverse |  |  |
| *CypA* | Cyclophilin A | NM_214353.1 | Pig | CCCACCGTCTTCTTCGACAT | Forward | 92 | [1] |
|  |  |  |  | TCTGCTGTCTTTGGAACTTTGTCT | Reverse |  |  |
| *ACTB* | β-actin | NM_213978.1 | Pig | GGACTTCGAGCAGGAGATGG | Forward | 230 | [1] |
|  |  |  |  | GCACCGTGTTTGCGTAGAGG | Reverse |  |  |
| *HPRT-1* | Hypoxanthine phosphoribosyl transferase 1 | NM_001032376.2 | Pig | TGGAAAGAATGTCTTGATTGTTGAAG | Forward | 93 | [1] |
|  |  |  |  | ATCTTTGGATTATGCTGCTTGACC | Reverse |  |  |
| *RPL 32* | Ribosomal Protein L32 | NM_001001636 | Pig | TGCTCTCAGACCCCTTGTGAAG | Forward | 106 | [1] |
|  |  |  |  | TTTCCGCCAGTTCCGCTTA | Reverse |  |  |

1. Taranu I, H.M., Gras MA, Pistol GC, Lefter N, Palade M, Ropota M, Chedea VS, Marin DE, *Assessment of the effect of grape seed cake inclusion in the diet of healthy fattening-finishing pigs.* Journal of Animal Physiology and Animal Nutrition (Berlin), 2018. **102**(1): p. 12.

2. Taranu I, H.M., Gras MA, Pistol GC, Lefter N, Palade M, Ropota M, Chedea VS, Marin DE, *Effect of xenobiotic compounds from grape waste on liver function and oxidative status in pigs.* Archiva Zootechnica, 2017. **20**(2): p. 20.

3. Su G, Z.X., Wang Y, Chen D, Chen G, Li Y, He J, *ffects of plant essential oil supplementation on growth performance, immune function and antioxidant activities in weaned pigs.* Lipids in Health and Disease, 2018. **17**(1): p. 10.

4. Zheng P, S.Y., Tian Y, Zhang H, Yu B, He J, Mao X, Yu J, Luo Y, Luo J, Huang Z, Tian G, Chen H, Chen D, *Dietary Arginine Supplementation Affects Intestinal Function by Enhancing Antioxidant Capacity of a Nitric Oxide-Independent Pathway in Low-Birth-Weight Piglets.* The Journal of Nutrition, 2018. **148**(11): p. 9.

5. Belhaj A, D.L., Kerbaul F, Brimioulle S, Dewachter C, Naeije R, Rondelet B, *Heme oxygenase-1 and inflammation in experimental right ventricular failure on prolonged overcirculation-induced pulmonary hypertension.* PloS One, 2013. **25**(8).
