# Supplementary material for: Grape seed meal by-product is able to counteract oxidative stress induced by lipopolysaccharide and dextran sulphate in IPEC cells and piglets after weaning
Source: PLoS One. 2023 Apr 13;18(4):e0283607. doi: 10.1371/journal.pone.0283607 (PMC10101422; doi:10.1371/journal.pone.0283607)
Supplement: S2 Table — (DOCX) [file pone.0283607.s002.docx]

**Supplementary File S2. Table S2. *p*-values for the parameters analysed in the *in vitro* and *in vivo* experiments**

1. ***In vitro* experiment**

**A.1. *p*-values** **for** **the distribution of ROS (+) and ROS (-) cells in IPEC-1 cell population**.

| **ROS-** | | | | | | |
| --- | --- | --- | --- | --- | --- | --- |
|  | **Control** | **LPS** | **GSM** | **LPS + GSM** | **EGCG** | **LPS + EGCG** |
| **Control** | - | *0.000* | ns | ns | ns | 0.063 |
| **LPS** | *0.000* | *-* | *0.000* | *0.001* | *0.000* | *0.043* |
| **GSM** | ns | *0.000* | *-* | 0.056 | ns | *0.053* |
| **LPS + GSM** | ns | *0.001* | 0.056 | - | ns | ns |
| **EGCG** | ns | *0.000* | ns | ns | - | ns |
| **LPS + EGCG** | 0.063 | *0.043* | 0.053 | ns | ns | - |

| **ROS+** | | | | | | |  |
| --- | --- | --- | --- | --- | --- | --- | --- |
|  | **Control** | **LPS** | **GSM** | **LPS + GSM** | **EGCG** | **LPS + EGCG** | |
| **Control** | - | *0.000* | ns | ns | ns | 0.074 | |
| **LPS** | *0.000* | *-* | *0.000* | *0.001* | *0.000* | *0.050* | |
| **GSM** | ns | *0.000* | *-* | *0.054* | ns | 0.060 | |
| **LPS + GSM** | ns | *0.001* | 0.054 | - | ns | ns | |
| **EGCG** | ns | *0.000* | ns | ns | - | ns | |
| **LPS + EGCG** | 0.074 | ns | 0.060 | ns | ns | - | |

IPEC-1 cells were treated with LPS for 4 hours continuously and then with GSM extract or EGCG for another 24 hours. Control = untreated control cells; LPS = cells treated with 5 µg/ml LPS; GSM= cells treated with GSM (50 µg/mL); LPS + GSM= cells treated with LPS (5 µg/ml) and GSM (50 µg/ml); EGCG= cells treated with EGCG (50 µM); LPS + EGCG = cells treated with LPS (5 µg/ml) and EGCG (50 µM). (ns = unsignificant; p values < 0.050 were written in Italic).

**A.2. *p*-values** **for** **the gene expression in IPEC-1 cells**

| ***CAT* gene** | | | | | | | | | | | | | | | | | | | | |
| --- | --- | --- | --- | --- | --- | --- | --- | --- | --- | --- | --- | --- | --- | --- | --- | --- | --- | --- | --- | --- |
|  | | | **Control** | | | **LPS** | | | **GSM** | | | **LPS + GSM** | | | **EGCG** | | | **LPS + EGCG** | | |
| **Control** | | | - | | | *0.018* | | | ns | | | ns | | | ns | | | ns | | |
| **LPS** | | | *0.018* | | | *-* | | | *0.019* | | | *0.006* | | | 0.060 | | | *0.019* | | |
| **GSM** | | | ns | | | *0.019* | | | - | | | ns | | | ns | | | ns | | |
| **LPS + GSM** | | | ns | | | *0.006* | | | ns | | | - | | | ns | | | ns | | |
| **EGCG** | | | ns | | | 0.060 | | | ns | | | ns | | | - | | | ns | | |
| **LPS + EGCG** | | | ns | | | *0.019* | | | ns | | | ns | | | ns | | | - | | |
| ***SOD* gene** | | | | | | | | | | | | | | | | | | | | |
|  | **Control** | | | **LPS** | | | **GSM** | | | **LPS + GSM** | | | | **EGCG** | | | **LPS + EGCG** | | | |
| **Control** | - | | | *0.029* | | | *0.011* | | | ns | | | | ns | | | *0.027* | | | |
| **LPS** | *0.029* | | | - | | | *0.004* | | | 0.056 | | | | 0.056 | | | *0.016* | | | |
| **GSM** | *0.011* | | | *0.004* | | | - | | | ns | | | | ns | | | ns | | | |
| **LPS + GSM** | ns | | | 0.056 | | | ns | | | - | | | | ns | | | ns | | | |
| **EGCG** | ns | | | 0.056 | | | ns | | | ns | | | | - | | | ns | | | |
| **LPS + EGCG** | *0.027* | | | *0.016* | | | ns | | | ns | | | | ns | | | - | | | |
| ***GPx*** **gene** | | | | | | | | | | | | | | | | | | |  |  |
|  | **Control** | | | **LPS** | | | **GSM** | | | **LPS + GSM** | | | **EGCG** | | | **LPS + EGCG** | | |  |  |
| **Control** | - | | | *0.008* | | | ns | | | ns | | | ns | | | ns | | |  |  |
| **LPS** | *0.008* | | | - | | | *0.049* | | | *0.001* | | | ns | | | ns | | |  |  |
| **GSM** | ns | | | *0.049* | | | - | | | ns | | | ns | | | ns | | |  |  |
| **LPS + GSM** | ns | | | *0.001* | | | ns | | | - | | | ns | | | ns | | |  |  |
| **EGCG** | ns | | | ns | | | ns | | | ns | | | - | | | ns | | |  |  |
| **LPS + EGCG** | ns | | | ns | | | ns | | | ns | | | ns | | | - | | |  |  |
| ***eNOS*** **gene** | | | | | | | | | | | | | | | | | | | |  |
|  | | **Control** | | | **LPS** | | | **GSM** | | | **LPS + GSM** | | | **EGCG** | | | **LPS + EGCG** | | |  |
| **Control** | | - | | | *0.005* | | | ns | | | ns | | | *0.034* | | | ns | | |  |
| **LPS** | | *0.005* | | | - | | | *0.001* | | | *0.000* | | | *0.001* | | | *0.003* | | |  |
| **GSM** | | ns | | | *0.001* | | | - | | | ns | | | ns | | | ns | | |  |
| **LPS + GSM** | | ns | | | *0.000* | | | ns | | | - | | | ns | | | ns | | |  |
| **EGCG** | | *0.034* | | | *0.001* | | | ns | | | ns | | | - | | | ns | | |  |
| **LPS + EGCG** | | ns | | | *0.003* | | | ns | | | ns | | | ns | | | - | | |  |
| ***iNOS*** **gene** | | | | | | | | | | | | | | | | | | | | |
|  | **Control** | | | **LPS** | | | **GSM** | | | **LPS + GSM** | | | **EGCG** | | | **LPS + EGCG** | | | | |
| **Control** | - | | | *0.012* | | | *0.036* | | | ns | | | ns | | | ns | | | | |
| **LPS** | *0.012* | | | *-* | | | *0.002* | | | *0.014* | | | *0.019* | | | *0.004* | | | | |
| **GSM** | *0.036* | | | *0.002* | | | - | | | ns | | | ns | | | ns | | | | |
| **LPS + GSM** | ns | | | *0.014* | | | ns | | | - | | | ns | | | ns | | | | |
| **EGCG** | ns | | | *0.019* | | | ns | | | ns | | | - | | | ns | | | | |
| **LPS + EGCG** | ns | | | *0.004* | | | ns | | | ns | | | ns | | | - | | | | |
| ***Nrf2*** **gene** | | | | | | | | | | | | | | | | | | | |  |
|  | | | **Control** | | | **LPS** | | | **GSM** | | | **LPS + GSM** | | | **EGCG** | | | **LPS + EGCG** | |  |
| **Control** | | | - | | | *0.031* | | | *0.032* | | | ns | | | *0.028* | | | *0.035* | |  |
| **LPS** | | | *0.031* | | | *-* | | | *0.019* | | | *0.049* | | | *0.043* | | | *0.041* | |  |
| **GSM** | | | *0.032* | | | *0.019* | | | - | | | 0.072 | | | ns | | | ns | |  |
| **LPS + GSM** | | | ns | | | *0.049* | | | 0.072 | | | - | | | *0.023* | | | *0.038* | |  |
| **EGCG** | | | *0.028* | | | *0.043* | | | ns | | | *0.023* | | | - | | | ns | |  |
| **LPS + EGCG** | | | *0.035* | | | *0.041* | | | ns | | | *0.038* | | | ns | | | - | |  |
| ***NQO1*** **gene** | | | | | | | | | | | | | | | | | | | |  |
|  | **Control** | | | | **LPS** | | | **GSM** | | | **LPS + GSM** | | | **EGCG** | | | **LPS + EGCG** | | |  |
| **Control** | - | | | | *0.025* | | | ns | | | ns | | | *0.014* | | | *0.034* | | |  |
| **LPS** | *0.025* | | | | - | | | *0.006* | | | *0.009* | | | *0.000* | | | *0.003* | | |  |
| **GSM** | ns | | | | *0.006* | | | - | | | 0.066 | | | ns | | | ns | | |  |
| **LPS + GSM** | ns | | | | *0.009* | | | 0.066 | | | - | | | *0.004* | | | 0.067 | | |  |
| **EGCG** | *0.014* | | | | *0.000* | | | ns | | | *0.004* | | | *-* | | | *0.020* | | |  |
| **LPS + EGCG** | *0.034* | | | | *0.003* | | | ns | | | 0.067 | | | *0.020* | | | - | | |  |
| ***HO1*** **gene** | | | | | | | | | | | | | | | | | | | |  |
|  | | **Control** | | | **LPS** | | | **GSM** | | | **LPS + GSM** | | | **EGCG** | | | **LPS + EGCG** | | |  |
| **Control** | | - | | | *0.013* | | | ns | | | ns | | | ns | | | *0.018* | | |  |
| **LPS** | | *0.013* | | | *-* | | | *0.025* | | | *0.044* | | | *0.003* | | | *0.022* | | |  |
| **GSM** | | ns | | | *0.025* | | | - | | | ns | | | ns | | | 0.066 | | |  |
| **LPS + GSM** | | ns | | | *0.044* | | | ns | | | - | | | ns | | | ns | | |  |
| **EGCG** | | ns | | | *0.003* | | | ns | | | ns | | | - | | | *0.036* | | |  |
| **LPS + EGCG** | | *0.018* | | | *0.022* | | | 0.066 | | | ns | | | *0.036* | | | - | | |  |
| ***Keap1*** **gene** | | | | | | | | | | | | | | | | | | | |  |
|  | **Control** | | | **LPS** | | | **GSM** | | | **LPS + GSM** | | | **EGCG** | | | **LPS + EGCG** | | | |  |
| **Control** | - | | | *0.021* | | | ns | | | ns | | | ns | | | ns | | | |  |
| **LPS** | *0.021* | | | *-* | | | *0.012* | | | *0.011* | | | *0.014* | | | *0.019* | | | |  |
| **GSM** | ns | | | *0.012* | | | - | | | ns | | | 0.060 | | | *0.023* | | | |  |
| **LPS + GSM** | ns | | | *0.011* | | | ns | | | - | | | ns | | | ns | | | |  |
| **EGCG** | ns | | | *0.014* | | | 0.060 | | | ns | | | - | | | ns | | | |  |
| **LPS + EGCG** | ns | | | *0.019* | | | *0.023* | | | ns | | | ns | | | - | | | |  |

IPEC-1 cells were treated with LPS for 4 hours and then with GSM extract or EGCG for another 24 hours. Control = untreated control cells; LPS = cells treated with 5 µg/ml LPS; GSM= cells treated with GSM (50 µg/mL); LPS + GSM= cells treated with LPS (5 µg/ml) and GSM (50 µg/ml); EGCG= cells treated with EGCG (50 µM); LPS + EGCG = cells treated with LPS (5 µg/ml) and EGCG (50 µM). (ns = unsignificant; p values < 0.050 were written in Italic).

**A.3. *p*-values** **for** **the antioxidant activity in IPEC-1 cells**.

| **CAT activity** | | | | | | | | | | | | | | | | |  |  |  |
| --- | --- | --- | --- | --- | --- | --- | --- | --- | --- | --- | --- | --- | --- | --- | --- | --- | --- | --- | --- |
|  | **Control** | | | **LPS** | | | **GSM** | | | **LPS + GSM** | | | **EGCG** | | **LPS + EGCG** | |  |  |  |
| **Control** | - | | | *0.050* | | | 0.060 | | | 0.065 | | | ns | | ns | |  |  |  |
| **LPS** | *0.050* | | | *-* | | | *0.003* | | | *0.002* | | | *0.045* | | *0.012* | |  |  |  |
| **GSM** | 0.060 | | | *0.003* | | | *-* | | | ns | | | 0.072 | | ns | |  |  |  |
| **LPS + GSM** | 0.065 | | | *0.002* | | | ns | | | - | | | ns | | 0.073 | |  |  |  |
| **EGCG** | ns | | | *0.045* | | | 0.072 | | | ns | | | - | | 0.079 | |  |  |  |
| **LPS + EGCG** | ns | | | *0.012* | | | ns | | | 0.073 | | | 0.079 | | - | |  |  |  |
| **SOD activity** | | | | | | | | | | | | | | | | | | |  |
|  | | **Control** | | | **LPS** | | | **GSM** | | | **LPS + GSM** | | **EGCG** | | **LPS + EGCG** | | |  |  |
| **Control** | | - | | | *0.005* | | | *0.000* | | | *0.000* | | *0.000* | | *0.000* | | |  |  |
| **LPS** | | *0.005* | | | *-* | | | *0.000* | | | *0.000* | | *0.000* | | *0.000* | | |  |  |
| **GSM** | | *0.000* | | | *0.000* | | | *-* | | | ns | | *0.000* | | *0.001* | | |  |  |
| **LPS + GSM** | | *0.000* | | | *0.000* | | | ns | | | - | | *0.000* | | *0.000* | | |  |  |
| **EGCG** | | *0.000* | | | *0.000* | | | *0.000* | | | *0.000* | | *-* | | ns | | |  |  |
| **LPS + EGCG** | | *0.000* | | | *0.000* | | | *0.001* | | | *0.000* | | ns | | - | | |  |  |
| **Total antioxidant capacity (TAC)** | | | | | | | | | | | | | | | | | | | |
|  | | | **Control** | | | **LPS** | | | **GSM** | | | **LPS + GSM** | | **EGCG** | | **LPS + EGCG** | | | |
| **Control** | | | - | | | ns | | | *0.000* | | | *0.000* | | *0.029* | | *0.008* | | | |
| **LPS** | | | ns | | | - | | | *0.000* | | | *0.000* | | *0.008* | | *0.000* | | | |
| **GSM** | | | *0.000* | | | *0.000* | | | *-* | | | ns | | *0.000* | | *0.000* | | | |
| **LPS + GSM** | | | *0.000* | | | *0.000* | | | ns | | | - | | *0.000* | | *0.000* | | | |
| **EGCG** | | | *0.029* | | | *0.008* | | | *0.000* | | | *0.000* | | *-* | | ns | | | |
| **LPS + EGCG** | | | *0.008* | | | *0.000* | | | *0.000* | | | *0.000* | | ns | | - | | | |

IPEC-1 cells were treated with LPS for 4 hours and then with GSM extract or EGCG for another 24 hours. Control = untreated control cells; LPS = cells treated with 5 µg/ml LPS~~;~~ GSM= cells treated with GSM (50 µg/mL); LPS + GSM= cells treated with LPS (5 µg/ml) and GSM (50 µg/ml); EGCG= cells treated with EGCG (50 µM); LPS + EGCG = cells treated with LPS (5 µg/ml) and EGCG (50 µM). (ns = unsignificant; *p* values < 0.050 were written in Italic).

1. ***In vivo* experiment**

**B.1. *p*-values** **for** **the ROS and TBARS levels in colon and mesenteric lymph nodes**

| **ROS (Reactive Oxygen Species)** | | | | | | | | | | | |
| --- | --- | --- | --- | --- | --- | --- | --- | --- | --- | --- | --- |
| **Colon** | | | | | | | | | | | |
|  | **Control** | | | **DSS** | | **GSM** | | **DSS+GSM** | | | |
| **Control** | - | | | *0.044* | | ns | | ns | | | |
| **DSS** | *0.044* | | | - | | 0.075 | | *0.021* | | | |
| **GSM** | ns | | | 0.075 | | - | | ns | | | |
| **DSS+GSM** | ns | | | *0.021* | | ns | | - | | | |
| **Mesenteric lymph nodes** | | | | | | | | | | | |
|  | **Control** | **DSS** | | | **GSM** | | | | **DSS+GSM** | |  |
| **Control** | - | *0.005* | | | ns | | | | *0.031* | |  |
| **DSS** | *0.005* | - | | | *0.007* | | | | *0.036* | |  |
| **GSM** | ns | *0.007* | | | - | | | | *0.009* | |  |
| **DSS+GSM** | *0.031* | *0.036* | | | *0.009* | | | | - | |  |
| **TBARS (Thiobarbituric acid reactive substances)** | | | | | | | | | | |  |
| **Colon** | | | | | | | | | | |  |
|  | **Control** | | **DSS** | | | | **GSM** | | | **DSS+GSM** |  |
| **Control** | - | | *0.000* | | | | ns | | | ns |  |
| **DSS** | *0.000* | | *-* | | | | *0.000* | | | *0.000* |  |
| **GSM** | ns | | *0.000* | | | | *-* | | | ns |  |
| **DSS+GSM** | ns | | *0.000* | | | | ns | | | - |  |
| **Mesenteric lymph nodes** | | | | | | | | | | |  |
|  | **Control** | | **DSS** | | | | **GSM** | | | **DSS+GSM** |  |
| **Control** | - | | *0.000* | | | | ns | | | ns |  |
| **DSS** | *0.000* | | *-* | | | | *0.000* | | | *0.000* |  |
| **GSM** | ns | | *0.000* | | | | *-* | | | ns |  |
| **DSS+GSM** | ns | | *0.000* | | | | ns | | | - |  |

Unchallenged and DSS-treated pigs were assigned for 30 days to a control diet (Control and DSS groups) or 8% GSM diet (GSM and DSS + GSM groups). At the end of the experiment. colon samples from all animals (n = 5) were collected and analysed for DNA oxidation and protein carbonyl content.). (ns = unsignificant; p values < 0.050 were written in Italic).

**B.2. *p*-values** **for** **the DNA oxidative damage and on protein carbonylation**

| **DNA oxidation (8-oxo dG)** | | | | | | | | | | | | |
| --- | --- | --- | --- | --- | --- | --- | --- | --- | --- | --- | --- | --- |
| **Colon** | | | | | | | | | | | | |
|  | | **Control** | | | **DSS** | | | | **GSM** | | | **DSS+GSM** |
| **Control** | | - | | | *0.041* | | | | ns | | | ns |
| **DSS** | | *0.041* | | | *-* | | | | 0.055 | | | *0.011* |
| **GSM** | | ns | | | 0.055 | | | | - | | | ns |
| **DSS+GSM** | | ns | | | *0.011* | | | | ns | | | - |
| **Mesenteric lymph nodes** | | | | | | | | | | | | |
|  | | **Control** | | | **DSS** | | | | **GSM** | | | **DSS+GSM** |
| **Control** | | - | | | *0.017* | | | | ns | | | ns |
| **DSS** | | *0.017* | | | *-* | | | | 0.056 | | | *0.034* |
| **GSM** | | ns | | | 0.056 | | | | - | | | ns |
| **DSS+GSM** | | ns | | | *0.034* | | | | ns | | | - |
| **Protein carbonyl content** | | | | | | | | | | | | |
| **Colon** | | | | | | | | | | | | |
|  | | | **Control** | | | **DSS** | | **GSM** | | **DSS+GSM** | | |
| **Control** | | | - | | | *0.045* | | ns | | ns | | |
| **DSS** | | | *0.045* | | | *-* | | *0.011* | | *0.023* | | |
| **GSM** | | | ns | | | *0.011* | | *-* | | ns | | |
| **DSS+GSM** | | | ns | | | *0.023* | | ns | | - | | |
| **Mesenteric lymph nodes** | | | | | | | | | | | | |
|  | **Control** | | | **DSS** | | | **GSM** | | | | **DSS+GSM** | |
| **Control** | - | | | *0.003* | | | ns | | | | *0.036* | |
| **DSS** | *0.003* | | | *-* | | | *0.017* | | | | *0.000* | |
| **GSM** | ns | | | *0.017* | | | *-* | | | | ns | |
| **DSS+GSM** | *0.036* | | | *0.000* | | | ns | | | | - | |

Unchallenged and DSS-treated pigs were assigned for 30 days to a control diet (Control and DSS groups) or 8% GSM diet (GSM and DSS + GSM groups). At the end of the experiment. colon samples from all animals (n = 5) were collected and analysed for DNA oxidation and protein carbonyl content.). (ns = unsignificant; p values < 0.050 were written in Italic).

**B.3. *p*-values** **for** **the antioxidant genes expression in colon and mesenteric lymph nodes:**

**B.3.a. Colon**

| ***CAT* gene** | | | | | | | | |
| --- | --- | --- | --- | --- | --- | --- | --- | --- |
|  | | **Control** | | **DSS** | | **GSM** | | **DSS+GSM** |
| **Control** | | - | | *0.015* | | ns | | ns |
| **DSS** | | *0.015* | | *-* | | *0.003* | | *0.002* |
| **GSM** | | ns | | *0.003* | | *-* | | ns |
| **DSS+GSM** | | ns | | *0.002* | | ns | | - |
| ***SOD* gene** | | | | | | | | |
|  | | **Control** | | **DSS** | | **GSM** | | **DSS+GSM** |
| **Control** | | - | | *0.001* | | 0.063 | | 0.069 |
| **DSS** | | *0.001* | | *-* | | *0.047* | | *0.049* |
| **GSM** | | 0.063 | | *0.047* | | *-* | | ns |
| **DSS+GSM** | | 0.069 | | *0.049* | | ns | | - |
| ***GPx* gene** | | | | | | | | |
|  | **Control** | | **DSS** | | **GSM** | | **DSS+GSM** | |
| **Control** | - | | 0.052 | | ns | | 0.070 | |
| **DSS** | 0.052 | | - | | 0.067 | | 0.071 | |
| **GSM** | ns | | 0.067 | | - | | ns | |
| **DSS+GSM** | 0.070 | | 0.071 | | ns | | - | |
| ***iNOS* gene** | | | | | | | | |
|  | **Control** | | **DSS** | | **GSM** | | **DSS+GSM** | |
| **Control** | - | | *0.000* | | ns | | *0.000* | |
| **DSS** | *0.000* | | *-* | | *0.050* | | *0.061* | |
| **GSM** | ns | | *0.050* | | *-* | | *0.000* | |
| **DSS+GSM** | *0.000* | | 0.061 | | *0.000* | | *-* | |
| ***eNOS* gene** | | | | | | | | |
|  | **Control** | | **DSS** | | **GSM** | | **DSS+GSM** | |
| **Control** | - | | *0.001* | | ns | | 0.070 | |
| **DSS** | *0.001* | | - | | *0.000* | | *0.030* | |
| **GSM** | ns | | *0.000* | | - | | ns | |
| **DSS+GSM** | 0.070 | | *0.030* | | ns | | - | |

Unchallenged and DSS-treated pigs were assigned for 30 days to a control diet (Control and DSS groups) or 8% GSM diet (GSM and DSS + GSM groups). At the end of the experiment. colon samples from all animals (n = 5) were collected and analysed for DNA oxidation and protein carbonyl content.). (ns = unsignificant; p values < 0.050 were written in Italic).

**B.3.b. Mesenteric lymph nodes**

| ***CAT* gene** | | | | | | | | |
| --- | --- | --- | --- | --- | --- | --- | --- | --- |
|  | | **Control** | | **DSS** | | **GSM** | | **DSS+GSM** |
| **Control** | | - | | *0.041* | | *0.012* | | ns |
| **DSS** | | *0.041* | | *-* | | *0.013* | | *0.045* |
| **GSM** | | *0.012* | | *0.013* | | *-* | | *0.006* |
| **DSS+GSM** | | ns | | *0.045* | | *0.006* | | - |
| ***SOD* gene** | | | | | | | | |
|  | | **Control** | | **DSS** | | **GSM** | | **DSS+GSM** |
| **Control** | | - | | *0.005* | | *0.038* | | ns |
| **DSS** | | *0.005* | | *-* | | *0.030* | | *0.012* |
| **GSM** | | *0.038* | | *0.030* | | *-* | | *0.031* |
| **DSS+GSM** | | ns | | *0.012* | | *0.031* | | - |
| ***GPx* gene** | | | | | | | | |
|  | **Control** | | **DSS** | | **GSM** | | **DSS+GSM** | |
| **Control** | - | | 0.076 | | ns | | ns | |
| **DSS** | 0.076 | | - | | ns | | ns | |
| **GSM** | ns | | ns | | - | | ns | |
| **DSS+GSM** | ns | | ns | | ns | | - | |
| ***iNOS* gene** | | | | | | | | |
|  | **Control** | | **DSS** | | **GSM** | | **DSS+GSM** | |
| **Control** | - | | *0.036* | | ns | | ns | |
| **DSS** | *0.036* | | *-* | | *0.042* | | *0.015* | |
| **GSM** | ns | | *0.042* | | *-* | | ns | |
| **DSS+GSM** | ns | | *0.015* | | ns | | *-* | |
| ***eNOS* gene** | | | | | | | | |
|  | **Control** | | **DSS** | | **GSM** | | **DSS+GSM** | |
| **Control** |  | | *0.010* | | ns | | *0.044* | |
| **DSS** | *0.010* | |  | | *0.021* | | *0.006* | |
| **GSM** | ns | | *0.021* | |  | | *0.038* | |
| **DSS+GSM** | *0.044* | | *0.006* | | *0.038* | |  | |

Unchallenged and DSS-treated pigs were assigned for 30 days to a control diet (Control and DSS groups) or 8% GSM diet (GSM and DSS + GSM groups). At the end of the experiment. colon samples from all animals (n = 5) were collected and analysed for DNA oxidation and protein carbonyl content.). (ns = unsignificant; p values < 0.050 were written in Italic).

**B.4. *p*-values** **for** **the antioxidant enzyme activity and on total antioxidant capacity in colon and mesenteric lymph nodes**

**B.4.a. Colon**

| **CAT activity** | | | | | | | | |
| --- | --- | --- | --- | --- | --- | --- | --- | --- |
|  | | **Control** | | **DSS** | | **GSM** | | **DSS+GSM** |
| **Control** | | - | | *0.044* | | ns | | ns |
| **DSS** | | *0.044* | | *-* | | *0.001* | | *0.001* |
| **GSM** | | ns | | *0.001* | | *-* | | ns |
| **DSS+GSM** | | ns | | *0.001* | | ns | | - |
| **SOD activity** | | | | | | | | |
|  | | **Control** | | **DSS** | | **GSM** | | **DSS+GSM** |
| **Control** | | - | | *0.015* | | ns | | ns |
| **DSS** | | *0.015* | | *-* | | *0.015* | | *0.020* |
| **GSM** | | ns | | *0.015* | | *-* | | ns |
| **DSS+GSM** | | ns | | *0.020* | | ns | | - |
| **GPx activity** | | | | | | | | |
|  | **Control** | | **DSS** | | **GSM** | | **DSS+GSM** | |
| **Control** | - | | *0.019* | | ns | | ns | |
| **DSS** | *0.019* | | *-* | | *0.041* | | *0.044* | |
| **GSM** | ns | | *0.041* | | - | | ns | |
| **DSS+GSM** | ns | | *0.044* | | ns | | - | |
| **Total antioxidant capacity** | | | | | | | | |
|  | **Control** | | **DSS** | | **GSM** | | **DSS+GSM** | |
| **Control** | - | | *0.030* | | ns | | ns | |
| **DSS** | *0.030* | | *-* | | *0.050* | | *0.040* | |
| **GSM** | ns | | *0.050* | | - | | ns | |
| **DSS+GSM** | ns | | *0.040* | | ns | | - | |

Unchallenged and DSS-treated pigs were assigned for 30 days to a control diet (Control and DSS groups) or 8% GSM diet (GSM and DSS + GSM groups). At the end of the experiment. colon samples from all animals (n = 5) were collected and analysed for DNA oxidation and protein carbonyl content.). (ns = unsignificant; p values < 0.050 were written in Italic).

**B.4.a. Mesenteric lymph nodes**

| **CAT activity** | | | | | | | | |
| --- | --- | --- | --- | --- | --- | --- | --- | --- |
|  | | **Control** | | **DSS** | | **GSM** | | **DSS+GSM** |
| **Control** | | - | | *0.030* | | ns | | ns |
| **DSS** | | *0.030* | | *-* | | *0.006* | | *0.048* |
| **GSM** | | ns | | *0.006* | | *-* | | 0.070 |
| **DSS+GSM** | | ns | | *0.048* | | 0.070 | | - |
| **SOD activity** | | | | | | | | |
|  | | **Control** | | **DSS** | | **GSM** | | **DSS+GSM** |
| **Control** | | - | | *0.002* | | ns | | 0.061 |
| **DSS** | | *0.002* | | *-* | | *0.000* | | *0.007* |
| **GSM** | | ns | | *0.000* | | *-* | | 0.067 |
| **DSS+GSM** | | 0.061 | | *0.007* | | 0.067 | | - |
| **GPx activity** | | | | | | | | |
|  | **Control** | | **DSS** | | **GSM** | | **DSS+GSM** | |
| **Control** |  | | *0.000* | | ns | | ns | |
| **DSS** | *0.000* | |  | | *0.000* | | 0.079 | |
| **GSM** | ns | | *0.000* | |  | | *0.001* | |
| **DSS+GSM** | ns | | 0.079 | | *0.001* | |  | |
| **Total antioxidant capacity** | | | | | | | | |
|  | **Control** | | **DSS** | | **GSM** | | **DSS+GSM** | |
| **Control** |  | | *0.005* | | ns | | ns | |
| **DSS** | *0.005* | |  | | *0.001* | | *0.024* | |
| **GSM** | ns | | *0.001* | |  | | ns | |
| **DSS+GSM** | ns | | *0.024* | | ns | |  | |

Unchallenged and DSS-treated pigs were assigned for 30 days to a control diet (Control and DSS groups) or 8% GSM diet (GSM and DSS + GSM groups). At the end of the experiment. colon samples from all animals (n = 5) were collected and analysed for DNA oxidation and protein carbonyl content.). (ns = unsignificant; p values < 0.050 were written in Italic).

**B.5. *p*-values** **for** **the signalling gene expression in colon and mesenteric lymph nodes**

**B.5.a Colon**

| **Nrf 2** | | | | |
| --- | --- | --- | --- | --- |
|  | **Control** | **DSS** | **GSM** | **DSS+GSM** |
| **Control** | - | *0.044* | ns | *0.047* |
| **DSS** | *0.044* | *-* | *0.010* | *0.013* |
| **GSM** | ns | *0.010* | *-* | *0.043* |
| **DSS+GSM** | *0.047* | *0.013* | *0.043* | *-* |
| **Keap 1** | | | | |
|  | **Control** | **DSS** | **GSM** | **DSS+GSM** |
| **Control** | *-* | *0.034* | *ns* | *ns* |
| **DSS** | *0.034* | *-* | *0.028* | *0.031* |
| **GSM** | *ns* | *0.028* | *-* | *ns* |
| **DSS+GSM** | *ns* | *0.031* | *ns* | *-* |
| **HO1** | | | | |
|  | **Control** | **DSS** | **GSM** | **DSS+GSM** |
| **Control** | *-* | *0.002* | *0.059* | *0.048* |
| **DSS** | *0.002* | *-* | *0.001* | *0.045* |
| **GSM** | *0.059* | *0.001* | *-* | *0.013* |
| **DSS+GSM** | *0.048* | *0.045* | *0.013* | *-* |
| **NQO1** | | | | |
|  | **Control** | **DSS** | **GSM** | **DSS+GSM** |
| **Control** | - | *0.011* | ns | ns |
| **DSS** | *0.011* | *-* | *0.008* | *0.016* |
| **GSM** | ns | *0.008* | *-* | ns |
| **DSS+GSM** | ns | *0.016* | ns | - |

Unchallenged and DSS-treated pigs were assigned for 30 days to a control diet (Control and DSS groups) or 8% GSM diet (GSM and DSS + GSM groups). At the end of the experiment. colon samples from all animals (n = 5) were collected and analysed for DNA oxidation and protein carbonyl content.). (ns = unsignificant; p values < 0.050 were written in Italic).

**B.5.a. Mesenteric lymph nodes**

| **Nrf 2** | | | | |
| --- | --- | --- | --- | --- |
|  | **Control** | **DSS** | **GSM** | **DSS+GSM** |
| **Control** | - | *0.050* | *0.036* | 0.069 |
| **DSS** | *0.050* | *-* | *0.007* | *0.045* |
| **GSM** | *0.036* | *0.007* | *-* | *0.050* |
| **DSS+GSM** | 0.069 | *0.045* | *0.050* | *-* |
| **Keap 1** | | | | |
|  | **Control** | **DSS** | **GSM** | **DSS+GSM** |
| **Control** | *-* | *0.004* | ns | ns |
| **DSS** | *0.004* | *-* | *0.000* | *0.019* |
| **GSM** | ns | *0.000* | *-* | ns |
| **DSS+GSM** | ns | *0.019* | ns | *-* |
| **HO1** | | | | |
|  | **Control** | **DSS** | **GSM** | **DSS+GSM** |
| **Control** | *-* | *0.007* | *0.033* | *0.040* |
| **DSS** | *0.007* | *-* | *0.007* | *0.010* |
| **GSM** | *0.033* | *0.007* | *-* | ns |
| **DSS+GSM** | *0.040* | *0.010* | ns | *-* |
| **NQO1** | | | | |
|  | **Control** | **DSS** | **GSM** | **DSS+GSM** |
| **Control** | - | *0.005* | ns | *0.022* |
| **DSS** | *0.005* | *-* | ns | *0.015* |
| **GSM** | ns | ns | *-* | *0.010* |
| **DSS+GSM** | *0.022* | *0.015* | *0.010* | - |

Unchallenged and DSS-treated pigs were assigned for 30 days to a control diet (Control and DSS groups) or 8% GSM diet (GSM and DSS + GSM groups). At the end of the experiment. colon samples from all animals (n = 5) were collected and analysed for DNA oxidation and protein carbonyl content.). (ns = unsignificant; p values < 0.050 were written in Italic).

**B.6. *p*-values** **for** **the Nrf2 protein expression in colon and mesenteric lymph nodes**

**B.6.a Colon**

| **Total Nrf 2** | | | | |
| --- | --- | --- | --- | --- |
|  | **Control** | **DSS** | **GSM** | **DSS+GSM** |
| **Control** |  | *0.020* | ns | ns |
| **DSS** | *0.020* |  | *0.000* | *0.006* |
| **GSM** | ns | *0.000* |  | 0.069 |
| **DSS+GSM** | ns | *0.006* | 0.069 |  |
| **Cytoplasmic Nrf2** | | | | |
|  | **Control** | **DSS** | **GSM** | **DSS+GSM** |
| **Control** | - | *0.012* | ns | ns |
| **DSS** | *0.012* | *-* | *0.019* | *0.022* |
| **GSM** | ns | *0.019* | - | ns |
| **DSS+GSM** | ns | *0.022* | ns | - |
| **Nuclear Nrf2** | | | | |
|  | **Control** | **DSS** | **GSM** | **DSS+GSM** |
| **Control** | - | *0.040* | ns | ns |
| **DSS** | *0.040* | *-* | *0.033* | *0.050* |
| **GSM** | ns | *0.033* | - | ns |
| **DSS+GSM** | ns | *0.050* | ns | - |

Unchallenged and DSS-treated pigs were assigned for 30 days to a control diet (Control and DSS groups) or 8% GSM diet (GSM and DSS + GSM groups). At the end of the experiment. colon samples from all animals (n = 5) were collected and analysed for DNA oxidation and protein carbonyl content.). (ns = unsignificant; p values < 0.050 were written in Italic).

**B.6.a. Mesenteric lymph nodes**

| **Total Nrf 2** | | | | |
| --- | --- | --- | --- | --- |
|  | **Control** | **DSS** | **GSM** | **DSS+GSM** |
| **Control** | - | *0.004* | ns | 0.068 |
| **DSS** | *0.004* | *-* | *0.002* | *0.002* |
| **GSM** | ns | *0.002* | *-* | 0.059 |
| **DSS+GSM** | 0.068 | *0.002* | 0.059 | *-* |
| **Cytoplasmic Nrf2** | | | | |
|  | **Control** | **DSS** | **GSM** | **DSS+GSM** |
| **Control** | - | *0.012* | ns | ns |
| **DSS** | *0.012* | *-* | *0.019* | *0.022* |
| **GSM** | ns | *0.019* | - | ns |
| **DSS+GSM** | ns | *0.022* | ns | - |
| **Nuclear Nrf2** | | | | |
|  | **Control** | **DSS** | **GSM** | **DSS+GSM** |
| **Control** | - | *0.020* | ns | *0.050* |
| **DSS** | *0.020* | *-* | *0.017* | *0.043* |
| **GSM** | ns | *0.017* | - | ns |
| **DSS+GSM** | *0.050* | *0.043* | ns | - |

Unchallenged and DSS-treated pigs were assigned for 30 days to a control diet (Control and DSS groups) or 8% GSM diet (GSM and DSS + GSM groups). At the end of the experiment. colon samples from all animals (n = 5) were collected and analysed for DNA oxidation and protein carbonyl content.). (ns = unsignificant; p values < 0.050 were written in Italic).
