# Supplementary material for: Grape seed meal by-product is able to counteract oxidative stress induced by lipopolysaccharide and dextran sulphate in IPEC cells and piglets after weaning
Source: PLoS One. 2023 Apr 13;18(4):e0283607. doi: 10.1371/journal.pone.0283607 (PMC10101422; doi:10.1371/journal.pone.0283607)
Supplement: S1 Raw images — (PDF) [file pone.0283607.s003.pdf]

Gel 1 – Colon samples – total lysate – beta-actin

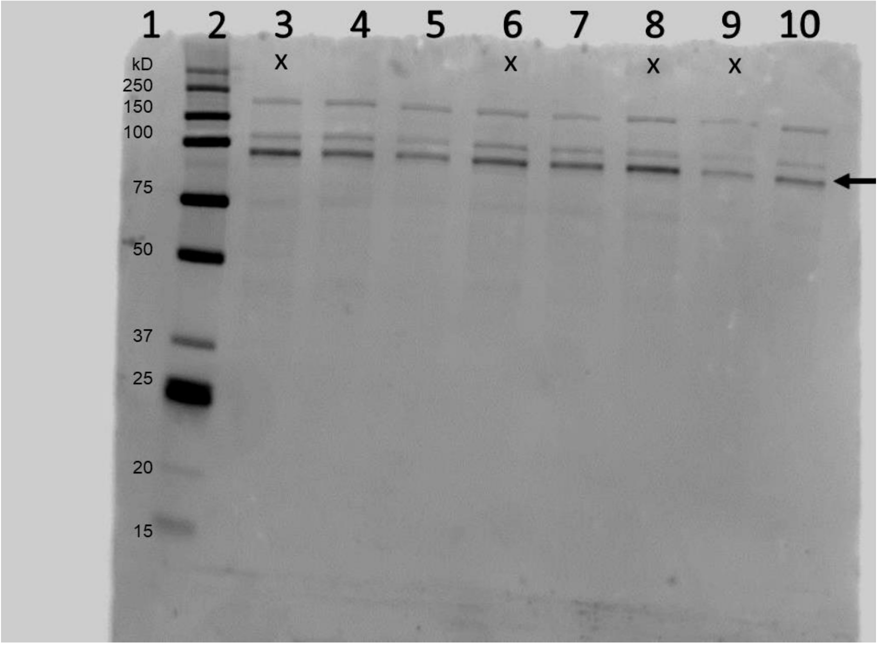

1- molecular weight marker

2 - Control 1

3 – control 2

4 – GSM 1

5 – GSM 2

6 – DSS +GSM 1

7 – DSS +GSM 2

8 – DSS 1

9 – DSS 2

10 – sample buffer

The image was captured with MicroChemi Imager, and was used to generate the blot image presented in Figure 2.B. in the manuscript. Lanes marked with X were not included in the final Figure.

Gel 2 – Colon samples – total lysate – Nrf2

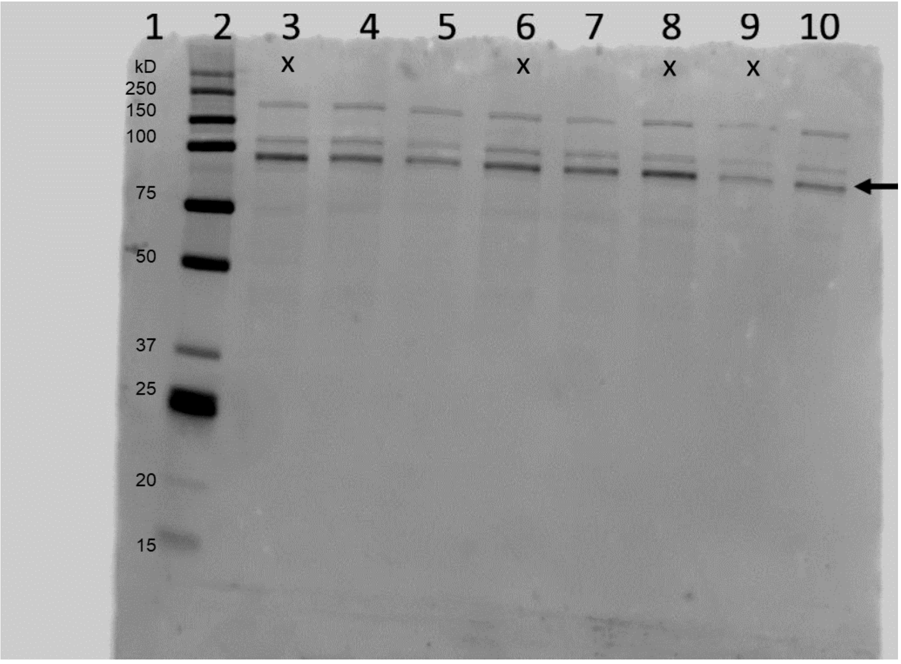

1- sample buffer

2 - molecular weight marker

3 - Control 1

4 – control 2

5 – GSM 1

6 – GSM 2

7 – DSS + GSM 1

8 – DSS + GSM 2

9 – DSS 1

10 – DSS 2

The image was captured with MicroChemi Imager, and was used to generate the blot image presented in Figure 2.B. in the manuscript. Lanes marked with X were not included in the final Figure.

Gel 3 – Mesenteric lymph nodes samples – total lysate – beta-actin

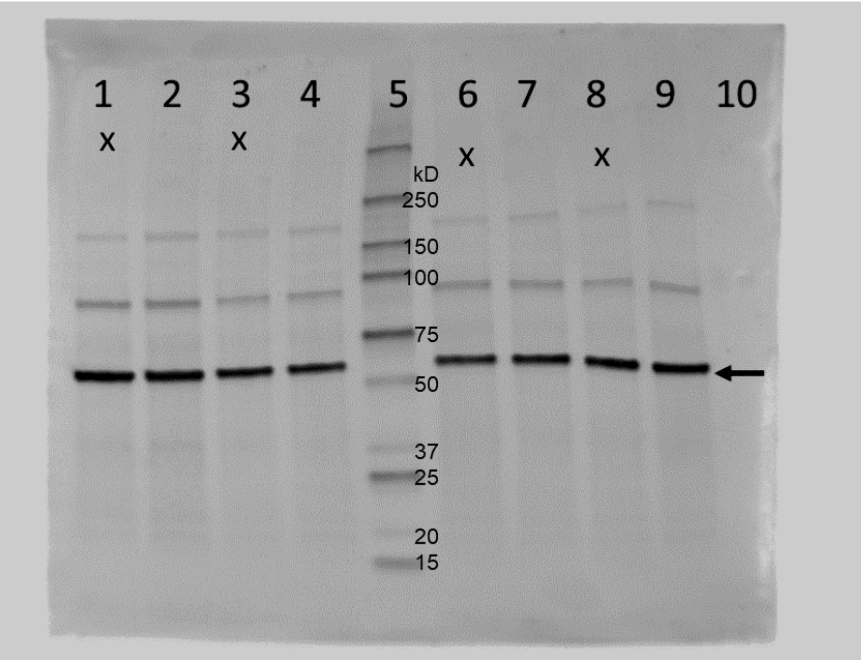

1 - Control 1

2 – control 2

3 – GSM 1

4 – GSM 2

5- molecular weight marker

6 – DSS 1

7 – DSS 2

8 – DSS + GSM 1

9 – DSS + GSM 2

10 – sample buffer

The image was captured with MicroChemi Imager, and was used to generate the blot image presented in Figure 2.B. in the manuscript. Lanes marked with X were not included in the final Figure.

Gel 4 – Mesenteric lymph nodes samples – total lysate – Nrf2

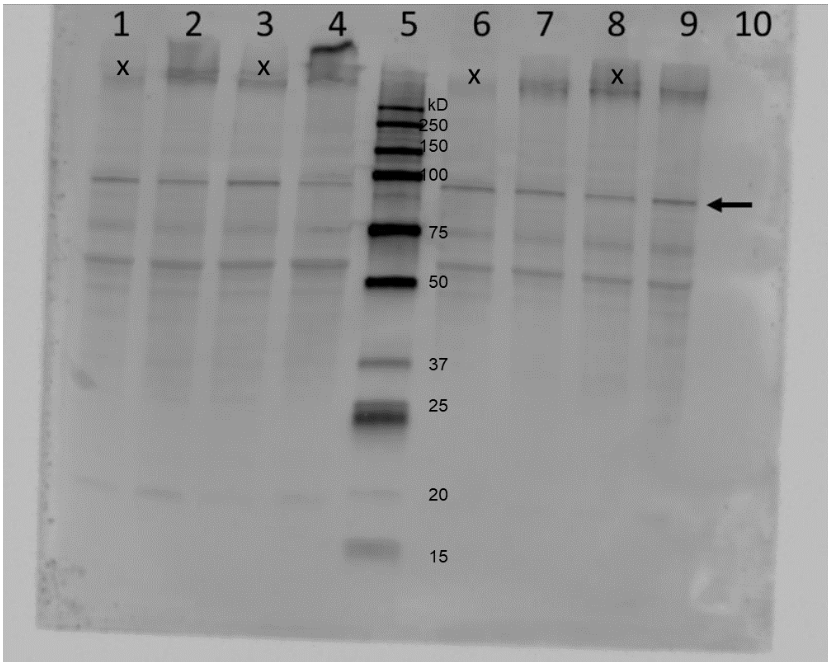

1 - Control 1

2 – control 2

3 – GSM 1

4 – GSM 2

5- molecular weight marker

6 – DSS 1

7 – DSS 2

8 – DSS + GSM 1

9 – DSS + GSM 2

10 – sample buffer

The image was captured with MicroChemi Imager, and was used to generate the blot image presented in Figure 2.B. in the manuscript. Lanes marked with X were not included in the final Figure.
